# Selective and predicable amine conjugation sites by kinetic characterization under excess reagents

**DOI:** 10.1038/s41598-021-00743-3

**Published:** 2021-10-27

**Authors:** Wei-Chun Huang, Li-Juan Huang, Liang-Sheng Hsu, Shih-Ting Huang, Wen-Ting Lo, Tzu-Fan Wang, Wei-Ting Sun, Win-Yin Wei, Ying-Shuan Lee, Shih-Hsien Chuang, Chao-Pin Lee, Ho-Hsuan Chou, Shu-Hui Chen

**Affiliations:** 1 Department of Chemistry, National Cheng Kung University, Tainan, 70101 Taiwan; 2 Department of Applied Chemistry, National Chiayi University, Chiayi, 600355 Taiwan; 3 Development Center for Biotechnology, Taipei, 11571 Taiwan; 4 Present Address: AIBIOS Co. Ltd., Tokyo, 106-0032 Japan

**Keywords:** Biochemistry, Biological techniques, Chemical biology, Chemistry

## Abstract

The site selectivity for lysine conjugation on a native protein is difficult to control and characterize. Here, we applied mass spectrometry to examine the conjugation kinetics of Trastuzumab-IgG (Her-IgG) and α-lactalbumin under excess linker concentration ([L]_0_) based on the modified Michaelis–Menten equation, in which the initial rate constant per amine (k_NH2_ = V_max/NH2_/*K*_*M*_) was determined by the maximum reaction rate (V_max/NH2_) under saturated accessible sites and initial amine–linker affinity (1/*K*_*M*_). Reductive amination (RA) displayed 3–4 times greater V_max/NH2_ and a different panel of conjugation sites than that observed for N-hydroxysuccinimide ester (NHS) chemistry using the same length of polyethylene glycol (PEG) linkers. Moreover, faster conversion power rendered RA site selectivity among accessible amine groups and a greater tunable range of linker/protein ratio for aldehyde-linkers compared to those of the same length of NHS-linkers. Single conjugation with high yield or poly-conjugations with site homogeneity was demonstrated by controlling [L]_0_ or gradual addition to minimize the [L]_0_/*K*_*M*_ ratio. Formaldehyde, the shortest aldehyde-linker with the greatest 1/*K*_*M*_, exhibited the highest selectivity and was shown to be a suitable probe to predict conjugation profile of aldehyde-linkers. Four linkers on the few probe-predicted hot spots were elucidated by kinetically controlled RA with conserved drug efficacy when conjugated with the payload. This study provides insights into controlling factors for homogenous and predictable amine bioconjugation.

## Introduction

Site-specific protein covalent modification is widely applied in many biomedical fields^1–3^. However, bioconjugation encounters numerous kinetic obstacles such as differential reaction kinetics of multiple active sites and competition induced by other residues on a protein. It is even more challenging to selectively modify more than one site of the same residue on a protein (poly-conjugation), which is a popular strategy for the development of antibody drug conjugates (ADCs) to increase drug efficacy^4,5^. Although unnatural amino acid^6^ or functional group incorporation^7^ by protein engineering has been reported for ADCs, chemical modification of natural amino acids on a native protein^8,9^ is the most straightforward approach, which saves time and is more economical.

Selective modification on native proteins may be achieved by reagent designs to impart the desired pathway or chemoselectivity. For example, affinity-guided 4-dimethylaminopyridine chemistry was designed to achieve selective protein acylation in tissue using a catalytic acyl transfer reaction^10^. A single-site labeling toward a specific His residue on native proteins was demonstrated exclusively through a linchpin-directed pathway^11^. A reversible intermolecular reaction places the “chemical linchpins” globally on all the accessible Lys residues to drive site selective labeling and ensure unaltered protein structure of the labeled protein^11^. Nevertheless, specific linkers may vary with different proteins and many reagents are not commercially available. On the other hand, non-specific labeling is more widely applicable.

Compared to other residues, lysine (Lys) conjugation is one of the most widely used non-specific conjugation strategies for natural amino acids. Lys can be modified efficiently by activated leaving groups, such as N-hydroxysuccinimidyl (NHS), sulfonyl chlorides, isocyanates, and isothiocyanates, as well as by reductive amination or 6π-aza electron cyclic reactions^12^. Due to the high natural abundance of Lys residues in proteins, heterogeneous conjugation numbers and sites are generated as revealed by mass spectrometry (MS) and bottom-up proteomics^13–15^, leading to some drawbacks such as suboptimal efficacy and a narrow therapeutic window of ADCs^16^. In particular, site-specificity which do not perturb immunoglobulin folding and assembly, or alter antigen binding is of critical to retain the antitumor efficacy of ADCs, while minimizing their systemic toxicity^17^ and conserving the antibody-linker stability^18^. Equal stoichiometry has been shown to selectively conjugate on unique hot spots created by molecular design using protein engineering^19^ or using site-specific linkers based on computer simulation^20^. However, as most conjugation reactions do not proceed to full completion^15^, the conjugation yield using equal or sub-equivalent amounts of reagents was extremely low for a natural protein.

Excess reagents are more commonly used for non-specific conjugation^12^, especially for poly-conjugation^13–15^. Considering reagent hydrolysis, kinetic models for protein modifications with NHS linkers was developed to predict the reagent concentration required to modify a protein at a given concentration to a specified number of modified amines per molecule^21^. For stable reagents such as aldehyde-linkers, aldehyde PEGylation kinetics was simulated using a numerically solved set of differential equations and showed that inactivation assumption is crucial for the overall simulation^22^. The data indicate that the reaction should be stopped before the highest mono-PEG concentration is reached in terms of selectivity and yield of mono-PEG. These models were shown to approximately match the results for the model protein and another protein with the same reagent under the same reaction conditions. Nevertheless, the site selectivity of modifications was not explored by these models.

Although abundant Lys sites exist on a protein, only a few active sites are freely accessible even in the presence of excess reagents and the reaction is likely to cease as all accessible sites are occupied. Selectivity among these accessible sites is of critical importance for bioconjugation. Such amine surface topology for aldehyde on a native IgG molecule has been recently characterized by formaldehyde labeling^23^. This is similar to enzyme catalytic motif to substrate in which the chemical conversion rate depends on the encounter rate between the substrate and the catalytic motif. Here, we intended to characterize Lys conjugation at residue or site level based on the enzyme kinetics model^24,25^ using MS and bottom-up proteomics to understand how the critical factors control the reaction. We compared the most commonly used NHS-linker with aldehyde-linker using reductive amination (RA). Moreover, in view of the impact of bioconjugation in ADC development, the characterization was conducted with the first generation ADC, Trastuzumab IgG (Her-IgG), which is a monoclonal antibody targeting HER2^26,27^ and contains 92 amine sites including 2 protein N-termini of the heavy and light chain. The model was further tested on another model protein, α-lactalbumin. We attempted to develop an integrated workflow to achieve a homogenous conjugation pattern based on a priori predicted hot spots using formaldehyde labeling^23^.

## Results

### Saturation kinetics model

The enzyme active sites bind substrate but remain un-changed throughout the catalysis. In bioconjugation, however, the amine sites on protein form covalent bonds with the linkers. Here we assume that the protein conformation remains un-changed throughout the conjugation reaction such that the reaction of individual amine sites is not much affected as more linkers react. Each site reaction can be viewed as an individual event which depends on local environments of respective amine groups such as their solvent accessibility and adjacent amino acids. We believe this assumption is generally applicable since most lysine residues are highly exposed and unlikely to alter the protein structure when conjugated unless intra-molecular interaction such as hydrogen bonding is generated. Thus, lysine conjugation under excess reagents involves contact frequency between the amine and the linker (binding affinity) and the subsequent chemical conversion. This is similar to enzyme–substrate reaction and may be modelled by enzyme kinetics^24,25^. The reaction begins with an initial bimolecular equilibrium between the protein (P) and the linker (L), and the product is the conjugate (P–L), which is converted from the complex (P*L):

1 $${\text{P}} + {\text{L}}\mathop{\rightleftarrows}\limits_{k_{-1}}^{k_{1}}{\text{P}}*{\text{L}}\mathop{\rightarrow}\limits^{{{\text{k}}_{2} }}{\text{P-L}}$$

**Figure 1:**
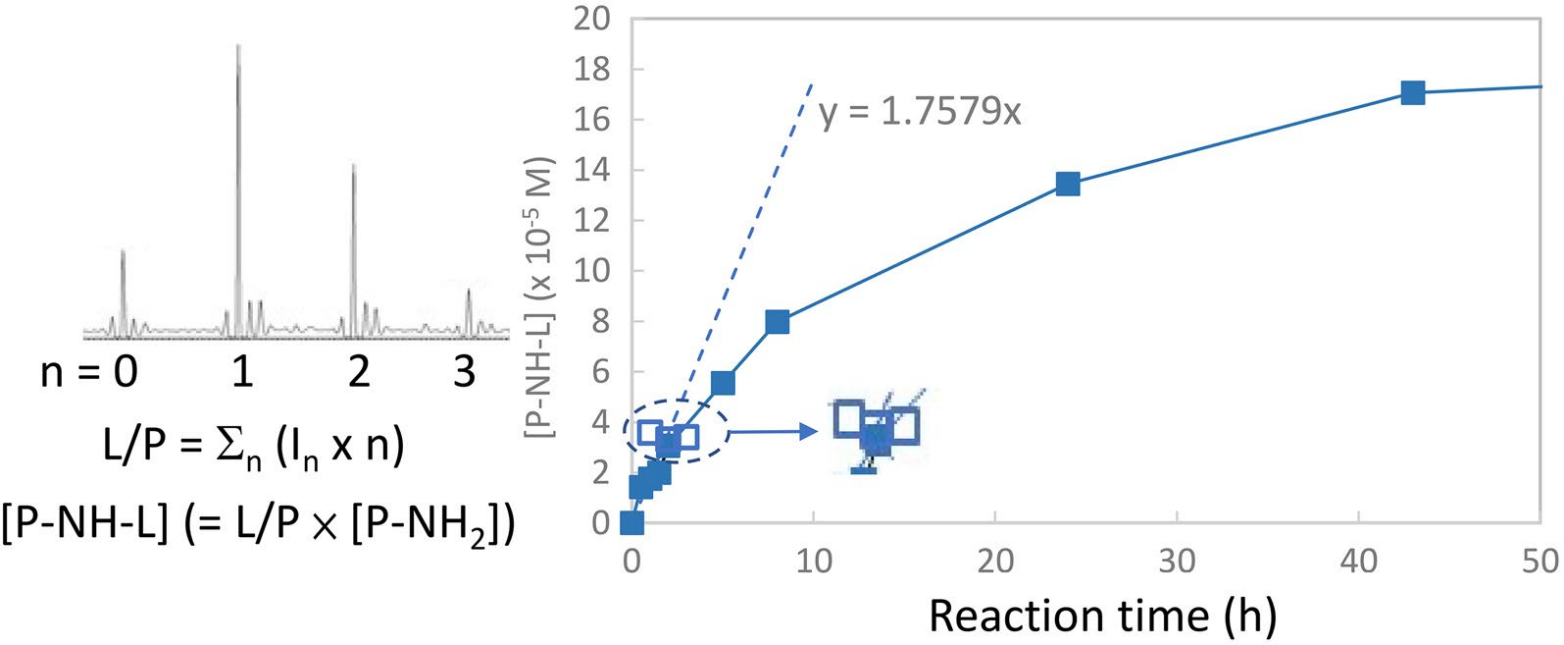
The initial rate (*v*_0/*NH2*_) measurement based on the slope (dashed line) of the time course study of the reaction of Her-IgG (5.73 μM) with AD-PEG-N_3_ (3 mM) (closed) and NHS-PEG-N_3_ (50 mM) (open). The products (P–L) were monitored as L/P ratios or the converted [P-HL-L] using intact protein measurement (left).

The product (P–L) was measured as the linker/protein (L/P) ratio by intact protein measurement using MS as shown in the left of Fig. 1.

The Michaelis–Menten equation^25^ was adopted to describe how the initial bimolecular reaction rate (*v*_0_) depends on the position of the binding equilibrium (k_1_/k_−1_) of the protein–linker complex (P*L) and the rate constant k_2_ of the intrinsic chemical conversion. Since amine group is the reaction site, the protein concentration [P] was expressed as free amine concentration [P-NH_2_] (= [P] × the number of amine groups on a protein. The initial reaction rate was measured as L/P per time (*v*_0_) or the linker conjugated amine concentration [P-NH-L] (= L/P × [P-NH_2_]) per time (*v*_0/*NH2*_) (Fig. 1). The modified Michaelis–Menten equation can be written in Eq. (2) in which V_max/NH2_ represents the maximum reaction rate per amine when all accessible amine sites are saturated, whereas k_2/NH2_ represents the rate constant of the intrinsic amine chemical conversion. *K*_*M*_ is the linker concentration at which *v*_0/*NH2*_ is half of V_max/NH2_. If k_2_ ≪ k_−1_, 1/*K*_*M*_ represents the P*L formation constant (k_1_/k_−1_) or the affinity of Lys for a particular linker.

2 $$\begin{gathered} v_{0/NH2} = \frac{{{\text{V}}_{{{\text{max}}/{\text{NH}}2}} \left[ {\text{L}} \right]}}{{K_{M} + \left[ {\text{L}} \right]}} \hfill \\ K_{M} = \, \left( {{\text{k}}_{{2}} + {\text{ k}}_{{ - {1}}} } \right)/{\text{k}}_{{1}} \sim \, \left( {{\text{k}}_{{ - {1}}} /{\text{k}}_{{1}} } \right){\text{ when k}}_{{2}} \ll {\text{ k}}_{{ - {1}}} \hfill \\ {\text{V}}_{{{\text{max}}/{\text{NH2}}}} = {\text{ k}}_{{{2}/{\text{NH2}}}} \left[ {{\text{P}} - {\text{NH}}_{{2}} } \right]_{0} \hfill \\ \end{gathered}$$

At relatively low linker concentrations, the L/P ratio is low and increases with the initial linker concentration [L]_0_ because of the increasing rate at which the accessible Lys group and linker molecules encounter one another. At high [L]_0_, the reaction rate approaches a theoretical maximum, V_max_, under which the accessible Lys sites on a protein are all occupied by linkers, resulting in a saturated L/P ratio.

The chemical conversion rate (k_2/NH2_) may constitute of a complex series of steps. For example, the RA reaction includes a reduction step. Using Her-IgG as a model, the conjugation yield increased with the borate concentration and reached a relatively constant level at high (> 0.04 M) concentration (Supplementary Figure S1). Thus, the k_2/NH2_ value would also reach a relatively constant level if the reducing agent is high enough. Throughout this study, borate reagent was added to the protein solution ([P-NH_2_]_0_ = 5.73 μM) at a final concentration of 0.158 M (dashed line in Figure S1). Therefore, the RA versus NHS reaction under different [L]_0_ or reaction time could be compared.

As shown in the right of Fig. 1, the *v*_0_ or *v*_0/*NH2*_ value was determined from the initial slope of the time course study of the Her-IgG conjugation reaction with the linkers, 2-(2-(2-(2-Azidoethoxy)ethoxy)ethoxy)acetaldehyde (AD-PEG-N_3_) or 2,5-Dioxopyrrolidin-1-yl-2-(2-(2-(2-Azidoethoxy)ethoxy)ethoxy)acetate (NHS-PEG-N_3_) (Fig. 1) or formaldehyde in slightly acidic (pH 5.8) and low temperature (8 °C) conditions, under which the protein remained native and the reaction rate was expected to be slower. According to CD spectroscopy measurements (Supplementary Figure S2), the secondary protein structure was confirmed to be unmodified by excess borate reducing agents, slightly acidic buffer, or low temperature. Acidic condition and lower temperature were also concluded to yield a higher selectivity by the previous study. RA reaction using AD-PEG-N_3_ continued up to > 50 h (Fig. 1); NHS reaction using NHS-PEG-N_3_, however, ended in 1–2 h (the enlargement of Fig. 1), which can be attributed to NHS hydrolysis in aqueous solution^21,28^.

### Modified Michaelis–Menten plots

**Figure 2:**
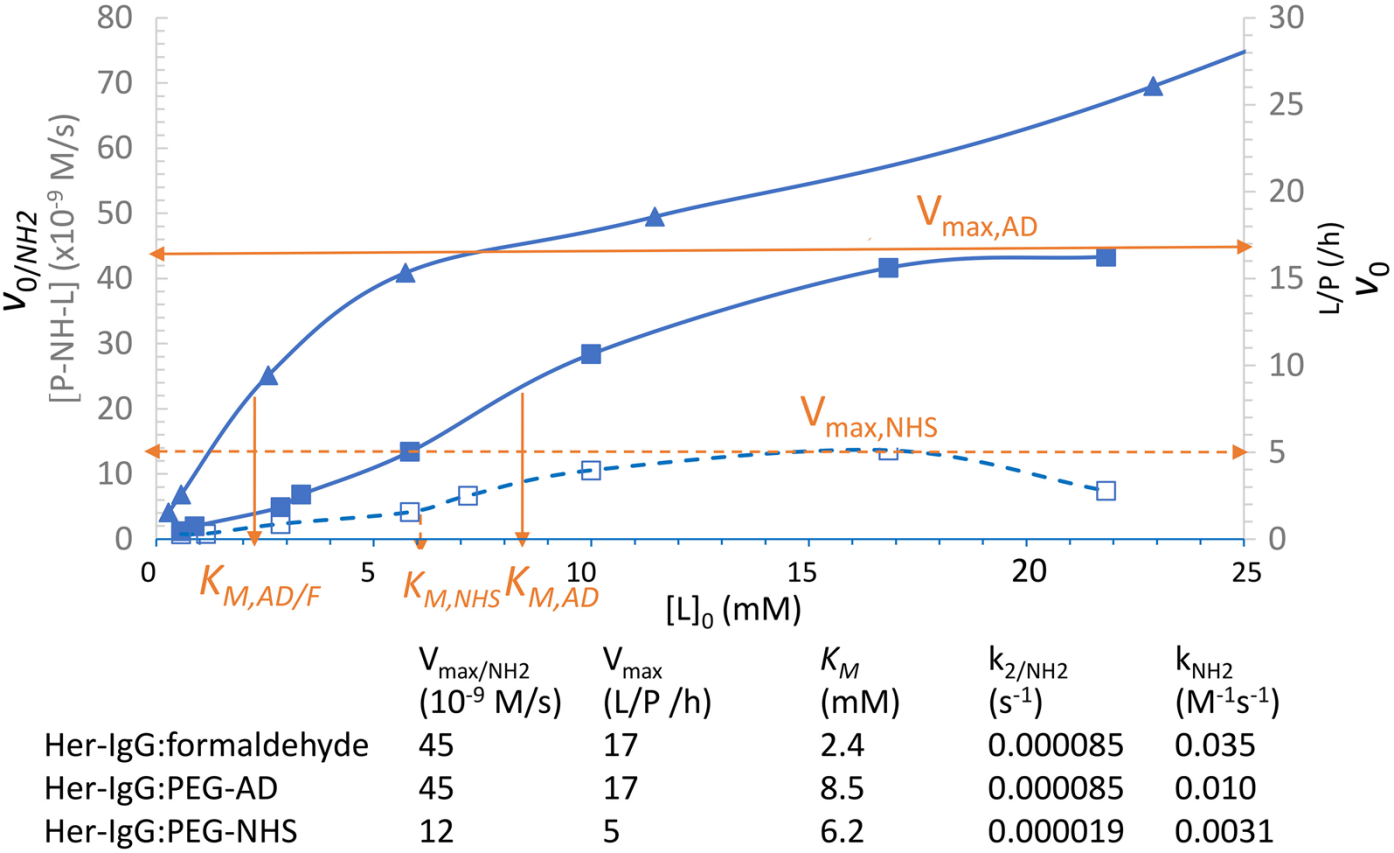
Modified Michaelis–Menten plots for bioconjugation of Her-IgG (5.73 μM) with formaldehyde (triangle) or AD-PEG-N_3_ (closed square) by RA versus with NHS-PEG-N_3_ (open square). The determined parameters from the plot were indicated in the bottom.

As shown in Fig. 2, the *v*_0/NH2_ or *v*_0_ value, measured as [P-NH-L]/s (left Y-axis) or L/P/h (right Y-axis), increased with [L]_0_. Formaldehyde (triangle) reached a sigmoidal plateau and continued to increase with a smaller slope. The AD-PEG-N_3_ conjugation (closed square) reached a similar plateau V_max/NH2_ (~ 47 × 10^–9^ M/s or 17 L/P/h), but no significant change was observed beyond that point. The NHS-PEG-N_3_ conjugation (open square) also reached a V_max/NH2_ (~ 11 × 10^–9^ M/s), which was observed to be approximately 4–5 times lower than the V_max/NH2_ of AD-PEG-N_3_; no significant change was observed beyond this level as well. These results indicate that bio-conjugation follows the saturation model as long as the linker is long enough like AD-PEG-N_3_ or NHS-PEG-N_3_.

However, formaldehyde still exhibited an intermediate maximum rate comparable to that of V_max/NH2_ (~ 45 × 10^–9^ M/s) of AD-PEG-N_3_, indicating a similar k_2/NH2_ (= V_max/NH2_/[P-NH_2_]_0_) value as same chemistry (RA) was used. It is also likely to exist a similar panel of conjugation sites for formaldehyde and AD-PEG-N_3_ linker. Beyond saturation (*v*_0_ > V_max_), formaldehyde was still able to contact the less accessible amine sites on Her-IgG, but the relatively bulky AD-PEG-N_3_ or NHS-PEG-N_3_ could not. Moreover, formaldehyde has the greatest amine affinity (1/*K*_*M*_) as it is the shortest aldehyde linker. This was also evidenced by two conjugated formaldehyde molecules per site (dimethyl labeling)^23^ versus only one PEG linker per site detected here.

Under *v*_0/*NH2*_ < V_max/NH2_ of Fig. 2_,_ Eq. (2) can be rewritten as an initial bimolecular reaction (Eq. 3).

3 $$\begin{gathered} v_{0/NH2} = {\text{ k}}_{{{2}/{\text{NH2}}}} /K_{M} \left[ {{\text{P}} - {\text{NH}}_{{2}} } \right]_{0} \left[ {\text{L}} \right]_{0} = {\text{ k}}_{{{\text{NH2}}}} \left[ {{\text{P}} - {\text{NH}}_{{2}} } \right]_{0} \left[ {\text{L}} \right]_{0} \hfill \\ {\text{k}}_{{{\text{NH2}}}} = {\text{ k}}_{{{2}/{\text{NH2}}}} /K_{M} \hfill \\ \end{gathered}$$

Since the [P-NH_2_]_0_ was kept constant (5.73 μM), the second order reaction (Eq. 3) can be simplified as pseudo first order reaction which linear range represents a kinetically tunable L/P ratios or conjugation levels by modulating [L]_0_ or reaction time. A greater V_max/NH2_ indicates a greater tunable range, whereas a greater k_NH2_ indicates faster reaction requiring fewer reagents. As summarized in the bottom of Fig. 2, AD-PEG-N_3_ had four times greater V_max/NH2_ value compared to NHS-PEG-N_3_, resulting in three times greater k_NH2_. Moreover, NHS reagents were almost completely hydrolyzed in 1–2 h (Fig. 1) and this inherent limitation associated with NHS chemistry could implicitly lower the overall reaction rate (k_NH2_). In contrast, the RA reagent was stable and lasted much longer. These features render RA more kinetic control to achieve the desired L/P ratio and selective conjugation sites by linker dosage or time with fewer reagents.

### Heat map generated via formaldehyde labeling

Since the initial binding affinity (1/*K*_*M*_) is an average of all accessible sites, formaldehyde labeling^23^ was applied to generate amine heat map (conjugation levels of each sites). It is important to be able to predict whether accessible hot spots on the intrinsic Her-IgG surface would block the antigen-binding domain prior to linker or payload conjugation. As shown in Fig. 2, formaldehyde has the highest V_max/NH2_ (comparable to AD-PEG-N_3_) as well as the highest amine affinity (1/*K*_*M*_), indicating a fast reaction and high selectivity. The heat map (Supplementary Figure S3) generated by 1-h reaction of formaldehyde (ranging from 0.29 to 45.84 mM) with Her-IgG (5.73 μM) indicated the most and second most accessible amine sites were the N-termini of the heavy (E1) and light (D1) chains, respectively. They were also observed to be the only two main conjugation sites (conjugation level > 20%) under low [L]_0_ (= 2.4 mM). Since Her-IgG consists of two heavy and two light chains, this corresponded to an L/P ratio ~ 4 calculated as twice the sum of the conjugation level/site. According to a previous report, these two N-termini are not in the antigen binding sites of Her-IgG^27^, indicating that the conjugated Her-IgG can be used to develop ADCs. The heat map (Figure S3) suggested higher site homogeneity under [L]_0_ < *K*_*M*_ (or [L]_0_/*K*_*M*_ < 1) and the conjugation sites became highly heterogeneous when [L]_0_ > *K*_*M*_.

### Linker conjugation profile

**Figure 3:**
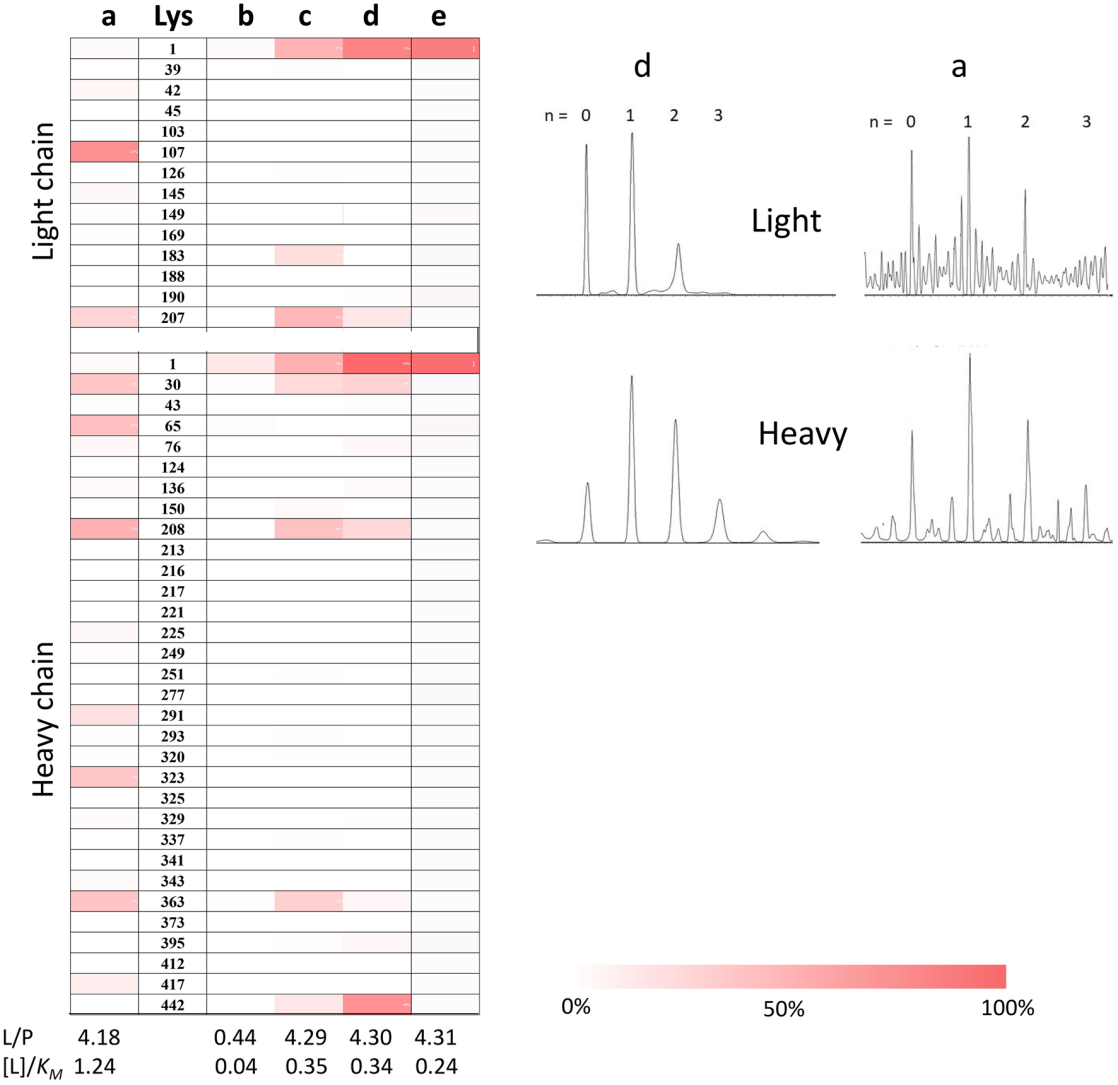
Conjugation levels for Lys sites of Her-IgG (100 μg) (left) generated by reaction with a: NHS-PEG-N_3_ (7.71 mM) for 1 h, b: AD-PEG-N_3_ (0.30 mM) for 1 h, c: AD-PEG-N_3_ (3.0 mM) for 1 h, d: AD-PEG-N_3_ (2.89 mM) for 4 h with gradual addition, and e: formaldehyde (0.57 mM) for 1 h. The L/P (~ 4) and L/*K*_*M*_ ratios are indicated in the bottom of the map. The deconvoluted spectra of the light chain (right top) and heavy chain (right bottom) of “d” and “a” shown in the left.

Since the L/P ratio of ~ 4 is an optimum drug load for developing ADCs, the profile of conjugation sites generated by 1-h reaction with NHS-PEG-N_3_ and AD-PEG-N_3_ to achieve an L/P ~ 4 were identified using bottom-up proteomics under full sequence coverage of Her-IgG (Supplementary Table S1) and compared with the formaldehyde heat map. As shown in column a of Fig. 3, while 7.71 mM of [NHS-PEG-N_3_]_0_ was still within the linear range (*v*_0_ < V_max/NH2_ in Fig. 2), the value was greater than *K*_*M*_ ([L]_0_/*K*_*M*_ = 1.24). The resulting conjugation sites were highly heterogeneous, including K107 (light), K207 (light), K30 (heavy), K65 (heavy), K208 (heavy), K291 (heavy), K323 (heavy), and K363 (heavy), all of which showed > 20% conjugation levels. However, it was not possible to achieve an L/P ratio of ~ 4 by using a lower linker dosage for NHS chemistry due to its inherent low V_max_ value or by using a longer reaction time due to hydrolysis of NHS reagents. It is noted that these identified NHS conjugation sites were not accessible sites predicted by formaldehyde labeling (Figure S3). In contrast, some significant conjugation sites for NHS chemistry, such as K107 (light), K30 (heavy), and K363 (heavy), were less accessible sites based on the formaldehyde heat map (Figure S3), indicating that the preferential sites vary with the head groups of the linkers.

For RA (column b of Fig. 3), a low dosage (0.3 mM) of AD-PEG-N_3_ ([L]_0_/*K*_*M*_ = 0.035) yielded an L/P ratio of ~ 0.44, and the N-terminus of the heavy chain was the only site with > 10% conjugation level, which was consistent with it being the predicted hot spot. When the AD-PEG-N_3_ concentration was increased to 3 mM with [L]_0_/*K*_*M*_ = 0.35 to reach an L/P ratio of ~ 4.29, K207 (light), K208 (heavy), and K363 (heavy) were also significantly (> 20%) conjugated in addition to the N-termini of the heavy and light chains (column c). These sites were also identified as accessible sites based on formaldehyde labeling (Figure S3). Apparently, RA yielded a similar panel of conjugation sites using either formaldehyde or AD-PEG-N_3_, which is, however, different from that obtained by NHS chemistry even with the same length of linkers. Moreover, formaldehyde with a higher initial binding affinity (1/*K*_*M*_) yielded a higher site specificity for the final conjugate (column b of Fig. 3) compared to that of AD-PEG-N_3_ (column c of Fig. 3).

### Kinetically controlled RA

As shown in Fig. 3, the site specificity of AD-PEG-N_3_ (column b and c) is higher than that of NHS-PEG-N_3_ (column a) regardless of comparable binding affinity of the two PEG linkers (bottom of Fig. 2), indicating the site selectivity can be driven by a faster chemical conversion rate. To improve the site selectivity for AD-PEG-N_3_, the kinetics may be manipulated in a time-dependent manner via gradual or fractional addition^29^. A 4-h gradual addition of 2.89 mM AD-PEG-N_3_ yielded an L/P ratio of ~ 4.30 (column d in Fig. 3), with significant conjugation (> 20% conjugation level) on the two N-termini and relatively less conjugation on K442 (heavy). Compared to the 1-h direct RA reaction (column c), the conjugation level of these three sites increased at the expense of the less accessible K207 (light), K208 (heavy), and K363 (heavy), in which conjugation levels decreased upon gradual addition.

Under L/P ratio ~ 4, the homogeneity of formaldehyde labeling (column e of Fig. 3 or Figure S3) was the highest due to the highest affinity. Although AD-PEG-N_3_ has a lower amine affinity (1/*K*_*M*_), focused conjugation sites can still be achieved by minimizing the [L]_0_/*K*_*M*_ ratio and gradual addition. In contrast, due to low V_max/NH2_ and low linker stability, such approaches are difficult to apply using NHS chemistry to achieve site homogeneity. The conjugation number profile (right of Fig. 3) was harder to tune by kinetic control. For L/P ~ 4, the profile was similar for the products yielded by using NHS chemistry with [L]_0_/*K*_*M*_ ratio = 1.24 (column d) and that by using RA with [L]_0_/*K*_*M*_ ratio = 0.35 (column a). However, the site homogeneity was much lower for NHS chemistry compared to that using RA.

### Model verification

**Figure 4:**
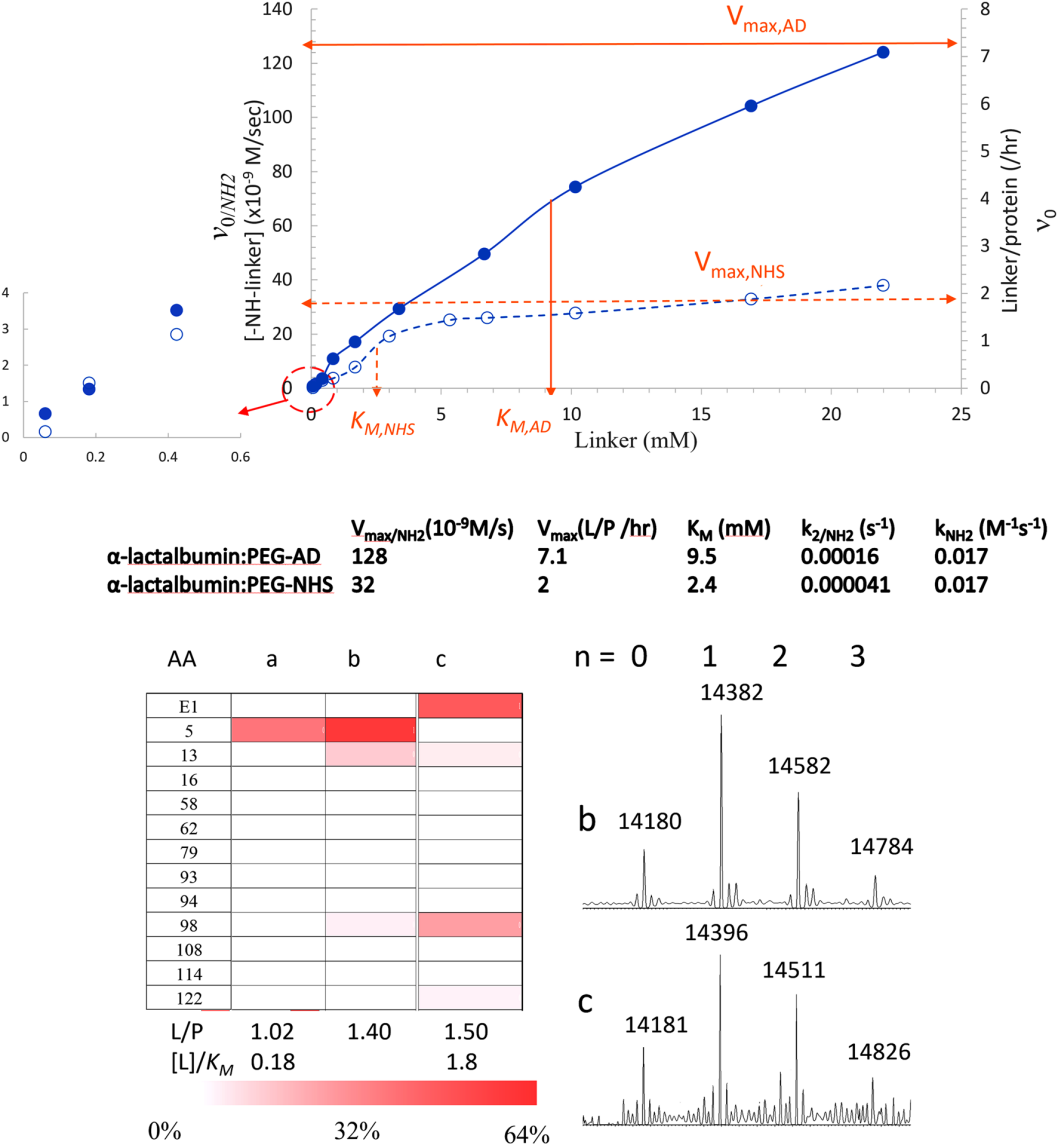
Comparability study of α-lactalbumin (100 μg). Modified Michaelis–Menten plots (top) for bioconjugation of AD-PEG-N_3_ (closed) versus NHS-PEG-N_3_ (open). The determined parameters from the plot were indicated in below. Distribution of the conjugation sites (bottom) obtained by reaction with a: AD-PEG-N_3_ (1.67 mM) for 1 h, b: AD-PEG-N_3_ (1.67 mM) for 3 h, and c: NHS-PEG-N_3_ (4.41 mM) for 1 h. The corresponding intact spectra of b and c were shown in the right.

As shown in Fig. 4, similar comparative parameters for RA versus NHS chemistry were also observed for another protein, α-lactalbumin. RA still showed a greater tunable range up to L/P ratio ~ 7 versus ~ 2 for NHS chemistry due to 4 times greater V_max/NH2_ (Fig. 4a). Moreover, a highly specific single conjugation (L/P ~ 1.02) on K5 of α-lactalbumin was achieved by a 1-h reaction with 1.67 mM AD-PEG-N_3_ with [L]_0_/*K*_*M*_ ~ 0.18 (Fig. 4b). Such high site selectivity for single conjugation by NHS reaction may be achieved by using a near-equal equivalence of linker dosage ([L]_0_/*K*_*M*_ ~ 0.18). However, the conjugation yield would be extremely low (L/P ~ 0.2) (circled inset of Fig. 4a). These results further confirm the applicability of the enzyme saturation model for bioconjugation and the low tunable range by NHS chemistry compared to RA, resulting in a low yield of selective single conjugation or low site heterogeneity of poly-conjugation when using kinetic controls.

### Cell viability measurements

**Figure 5:**
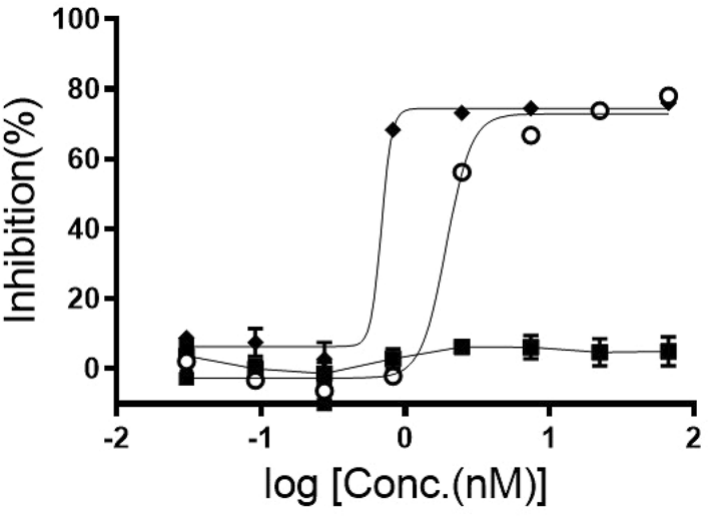
In vitro cancer cell killing activity of ADCs using SK-BR-3 cells. The IC_50_ values of ADC1 (open circle) and ADC2 (diamond) were 1.9 and 0.7 nM, respectively, against > 66.7 nM (square) for the control determined based on the data (mean ± SEM, n ≥ 2).

The AD-PEG-N_3_ conjugated Her-IgG was conjugated with the payload to generate ADC1 (DM1) and ADC2 (MMAF) through click chemistry via the azide functional group present at the other end of the linker (Supplementary Method). The drug efficacy of Her–DM1 was compared with that of the control without conjugation using Her2-targeting breast cancer cells (SK-BR-3). Dose response curves for ADCs using the breast cancer cell line SK-BR-3 were assessed using Cell Titer-Glo assay. As shown in Fig. 5, compared to Her, both ADC1 and ADC2 showed high potency and were able to kill HER2-overexpressing SK-BR-3 cells at low concentrations (< 2 nM). More experiments are required to examine whether higher homogeneity leads to higher drug efficacy. Although the second generation ADCs synthesized by conjugation to the reduced interchain disulfides of Her-IgG has been reported^30^, nonspecific lysine conjugation is still a valuable method for developing ADCs targeting many diseases.

## Discussion

A modified Michaelis–Menten equation was adopted to describe how the initial bimolecular reaction rate (k_NH2_) depends on the initial amine–linker binding affinity (~ 1/*K*_*M*_) and the maximum reaction rate (V_max/NH2_). The later depends on the chemical conversion rate (k_2/NH2_) and accessible amine sites. Site selectivity of the conjugates relies on the balance between the initial amine–linker binding affinity and the chemical conversion rate. Unlike selective conjugation, the amine–linker binding affinity is relatively weak and non-specific. Our data showed that fast chemical conversion can help to retain strongest binding sites before significant diffusion occurs. Moreover, fast overall reaction rate (k_NH2_ = k_2/NH2_/*K*_*M*_) renders fewer reagents to be consumed. Formaldehyde with all the three high values exhibited a fast reaction and high selectivity with fewer reagents; indicating formaldehyde is a suitable probe to predict conjugation reaction of aldehyde linkers.

As demonstrated by using AD-PEG-N_3_ linker, kinetic control via gradual or fractional addition to minimize the [L]_0_/*K*_*M*_ value can improve the site selectivity compared to that of direct addition while keeping the total added amount the same. This may be explained by the lower [L]_0_ at the instant of addition such that the amine–linker complex once formed can be timely converted to the product by subsequent chemical reactions. For lower affinity (1/*K*_*M*_) of protein–linker complex, a greater [L]_0_ may be used in order to keep adequate yields. Increasing the addition time, however, leads to greater chance of random interactions by diffusion as the case of increased conjugation on K442 (heavy) of Her-IgG. However, such kinetic control strategy cannot be widely applied for NHS chemistry due to slow kinetics and reagent hydrolysis. Although selective single conjugation of α-lactalbumin (top of Fig. 4) can be achieved by using a near-equal equivalence of NHS-linker, the conjugation yield was extremely low.

Compared to other models^21,22^, our model is unique in taking the initial site selectivity (1/*K*_*M*_) and saturation of accessible amine groups (V_max/NH2_) into consideration. Our data showed that a similar panel of conjugation sites was obtained for the same head group; increasing linker length (AD-PEG-N_3_) decreased the linker-amine affinity; while different head groups with the same linker length (NHS-PEG-N_3_) resulted in different panel of conjugation sites as revealed by bottom-up proteomics. Such results support the assumption that initial binding affinity of linkers plays a role in amine conjugation kinetics to affect site selectivity of the final products. Moreover, several similar observations were also revealed by the previous study for deriving linker conjugation model^21,22^. For aldehyde linkers^22^, inactivation reaction seemed to be essential for mathematical modeling. Moreover, inactivation only occurred in the presence of protein and should not be due to reagent hydrolysis^22^. Using our model, inactivation was described by V_max/NH2_ when all accessible amine groups are occupied by the linkers. Secondly, experimental conditions used to obtain the best selectivity were found not to result in highest conversion yield^22^. Here we proposed to minimize the [L]_0_/*K*_*M*_ value in order to balance the selectivity and conversion yield. Compared to the amine conjugation model for NHS-linkers^21^ which took linker hydrolysis into consideration, here multiple chemical conversion steps or reagent degradation steps were actually implied in the rate constant k_2/NH2_ or k_NH2_ of the current model. As shown, the greater k_2/NH2_ for AD-PEG-N_3_ linker compared to that of NHS-PEG-N_3_ linker is due to the use of relatively high concentration of reducing reagent and hydrolysis-free with the AD-PEG-N_3_ linker. Moreover, although the 1/*K*_*M*_ value is an average of all accessible sites, we believe the current model can be applied to describe specific conjugation which high initial affinity (1/*K*_*M*_) is associated with few selective sites by reagent design.

This study combines bottom-up proteomics and modified Michaelis–Menten model to provide insights on the site-selectivity and reaction kinetics. Fast chemical conversion rate of RA was shown to drive site selectivity among accessible amine groups on the protein. The greater V_max/NH2_ and k_NH2_ of AD-PEG-N_3_ compared to those of NHS-PEG-N_3_ allowed a relatively wide range of poly-conjugations focusing on few formaldehyde-predicted sites to be tuned by minimizing the [L]_0_/*K*_*M*_ ratio using fewer reagents in spite of slightly lower initial linker-amine affinity (~ 1/*K*_*M*_). Moreover, the drug efficacy was conserved when AD-PEG-N_3_ conjugated Her-IgG was conjugated with the payload. These insights should help in optimizing the reaction or designing new reagents for bio-conjugation.

## Method

### Materials

Sodium acetate (NaOAC), sodium bicarbonate (NaHCO_3_), formaldehyde-d2, sodium cyanoborohydride (NaBH_3_CN), ammonium hydroxide, dl-dithiothreitol (DTT), iodoacetamide (IAM), ammonium bicarbonate (NH_4_HCO_3_), guanidine hydrochloride (Gd-HCl), hydrochloric acid (HCl), sequence-grade chymotrypsin, α-lactalbumin type I, and endoproteinase Glu-C were purchased from Sigma-Aldrich (St. Louis, MO, USA). Sequence-grade PNGase F were purchased from Promega (Madison, WI, USA). LC–MS grade acetonitrile (ACN), methanol (MeOH), formic acid (FA), dimethylformamide (DMF) were purchased from Thermo Fisher Scientific Inc (Walthan, MA, USA). Water (ddH_2_O) was deionized to 18 MΩ by a Milli-Q system. Amicon centrifugal filters (10 or 30 kDa MWCO) were obtained from Millipore (Billerica, MA, USA). Human IgG (Trastuzumab) was from commercially available antibody drug product Her-IgG as the solution form (22 mg/mL). 2-(2-(2-(2-Azidoethoxy)ethoxy)ethoxy)acetaldehyde (AD-PEG-N_3_) and 2,5-Dioxopyrrolidin-1-yl 2-(2-(2-(2-azidoethoxy)ethoxy)ethoxy)acetate (NHS-PEG-N_3_) linkers were synthesized in house and their structures were confirmed by NMR and MS: ^1^**H-NMR** (**400 MHz**): *δ* 9.71 (s, 1H), 4.14 (s, 2H), 3.71–3.64 (m, 10H), 3.37 (t, *J* = 5.0 Hz, 2H) for NHS-PEG-N_3_; and ^1^**H-NMR** (**300 MHz**): *δ* 4.53 (s, 2H), 3.82–3.79 (m, 2H), 3.74–3.66 (m, 8H), 3.39 (t, J = 5.0 Hz, 2H), 2.34(s, 4H) for AD-PEG-N_3_.

### Conjugation reaction

Her-IgG or α-lactalbumin (100 μg) was dissolved in 100 μL sodium acetate buffer (100 mM, pH 5–6). For NHS reaction, the solution was added with NHS-PEG-N_3_ reagent (275 mM in 50% DMF) to yield a specified final concentration and subsequently, the solution was incubated at 8 °C in the dark for 1 h or longer time. For direct RA reaction, the solution was added with a small volume (less than 10 μL) of 6 M NaBH_3_CN at a specified molar ratio, vortexed for 5 min, and then added with 20 μL of AD-PEG-N_3_ reagent (275 mM in 50% methanol) to generate a specified molar ratio, followed by incubation at 8 °C in the dark for 1 h or longer time. For gradual addition of RA reaction, the protein solution was added with a small volume (less than 10 μL) of 6 M NaBH_3_CN at a specified molar ratio. After vortexing for 5 min, 20 μL of PEG-AD reagent (275 mM) was gradually added to the protein solution by a syringe pump at a constant flow rate to complete the addition in 1, 2, or 4 h at 8 °C in the dark.

### Payload conjugation to Her-IgG-PEG-AD

The Her-IgG-PEG-AD was conjugated with the anti-cancer drugs, DBCO-PEG4-DM1 (ADC1) and DBCO-MMAF (ADC2), respectively, through the azide functional group on the other end of the PEG-AD linker through click chemistry (Supplementary Method). To 200 μL protein solution (5.0 mg/mL) in buffer (25 mM MES Buffer pH 6.5) was slowly added with 15 equivalent of DBCO-linker-payload (10 mM in DMSO). The reaction mixture was stirred under argon at 37 °C for 20 h. After reaction, the solution was desalted and exchanged to PBS buffer (pH 7.4) using a 30 kDa molecular weight cutoff filter. The drug to antibody ratio was determined to be 2.93 for ADC 1 and 6.36 for ADC 2, respectively.

### Intact protein measurement

The method was described previously^23^. Briefly, the IgG solution (1 mg/mL) dissolved in ammonium bicarbonate (50 mM containing 6 M Gd-HCl, pH8) was added with PNGaseF (enzyme:protein = 1:100), incubated at 37 °C for 18 h to dissociate the glycan, and then reduced by DTT (10 mM) at 37 °C for 1 h to dissociate the heavy and light chain. The resulting protein solution was diluted with 0.2% FA to a final concentration of 0.5 µg/µL. A 3-μL volume of the samples was injected into an HPLC column (2.1 μm × 150 mm, 3.5 μm C4, BEH300, Waters Corporation, Milford, MA, USA), coupled online to a Q-TOF instrument (ACQUITY UPLC and Xevo G2-S QTof, Waters Corporation, Milford, MA, USA), and eluted at 40 °C using mobile phase A (0.1% FA) and mobile phase B (ACN in 0.1% FA) for a 30-min gradient: 0–5 min, 5% B, 5–17 min, 5% to 95% B, 17–23 min, 95% B, 23–26 min, 95% to 5% B, and 26–30 min, 5% B at a flow rate of 0.32 μL/min. The temperature of the desolvation gas and the source were set to 450 °C and 150 °C, respectively. The capillary and the cone voltage were set at 3.0 kV and 40 V, respectively, and the data (m/z 50–800 at a scan speed of 0.5 s/scan, and 500–3000 at a scan speed of 1.0 s/scan) were collected using the MassLynx 4.1 software. The acquired multiple charge profiles were de-convoluted using the MaxEnt 1 algorithm. The number of conjugated linker per protein (L/P) ratio was determined based on the weighted average of the conjugated linkers^23^.

$${\text{L/P }} = \Sigma_{{\text{n}}} ({\text{relative}}\;{\text{peak}}\;{\text{height}}\;{\text{or}}\;{\text{area}}\;(\% )_{{\text{n}}} \times {\text{ number}}\;{\text{of}}\;{\text{linker}}).$$

### Bottom-up proteomics

Following a previous method^23^, the protein solution (60 µg) was reduced with DTT and alkylated with IAM, followed by addition of PNGase-F (enzyme/protein ratio of 1:100) to remove N-glycans at Fc-IgG. Then, the solution was digested by chymotrypsin (enzyme:protein ratio of 1:25) at 37 °C for 18 h, dried, and then redissolved in ddH_2_O containing 0.1% FA. A volume of 1 μL of the resulting solution was injected onto Waters nanoACQUITY UPLC system equipped with aprecolumn (Waters, 0.180 mm × 20 mm, 5 μm C18) followed by a nanocolumn (Waters, 75 μm × 25 cm, 1.7 μm C18) in series coupled online to an LTQ-Orbitrap XL mass spectrometer. NanoLC-MS data were acquired using the data-dependent mode where one full scan with m/z 300–2000 in the LTQ-Orbitrap XL mass spectrometer (Thermo Fisher Scientific, San Jose, CA, USA) (R = 60,000 at m/z 400) at a scan rate of 30 ms/scan was followed by the five most intense peaks for fragmentation with a normalized collision energy value of 35% in the LTQ. A repeat duration of 30 s was applied to exclude the same m/z ions from being re-selected for fragmentation.

The raw data were converted to mgf peak lists and submitted to an in-house Mascot Sever (version 2.3, Matrix Science Ltd., London, UK) with default settings for Orbitrap to search against the in-house protein (α-Lactalbumin or Her-IgG) database. A mass tolerance of ± 10 ppm for the precursor ions and ± 0.8 Da for the product ions, chymotrypsin as enzyme specification, two allowable miss-cleavages, variable modifications by IAM (C; + 57.02 Da), Deamindation (N, Q; + 0.98 Da), Pyroglutamate (E at N termini of antibody; − 18.01 Da), AD-PEG-N_3_ (K; + 201.11 Da) and NHS-PEG-N_3_ (K; + 215.09 Da) were chosen and a Mascot probability score (P < 0.05) were used. Each identified sequence and conjugation site were manually inspected to confirm the match of unique fragments. The conjugation level for the individual sites was calculated by dividing the extracted ion intensity of the conjugated peptide by that of the sum of conjugated and non-conjugated peptides covering the same conjugation site^11–13,20^ with minor proportional adjustments based on the L/P ratio from intact protein measurement.

### Circular dichroism (CD) spectroscopy

Far-UV CD spectra were recorded as described previously^23^ using JASCO J-815 spectropolarimeter (JASCO Inc., Japan) equipped with a temperature-controlling liquid system. The spectra of Her-IgG (400 μg protein in 50 mM Tris–HCl Buffer containing 1 M NaBH_3_CN, pH 7.5) ranging from 200 to 260 nm were collected using cuvettes with a 1 mm path length, 0.1 nm resolution, 1.0-s response time, and 100 nm/min scanning speed. All the measurements were performed under a nitrogen flow. Each scan was repeated ten times to obtain an averaged value. The results were expressed as mean residue ellipticities [θ] in units of degrees cm^2^ dmol^−1^, which is defined as [θ] = 10θobs (lc) − 1, where θobs is the observed ellipticity in degrees, c is the concentration in moles per liter, and l is the length of the light path in centimeters.

### Cell viability assay

Breast cancer cell line SK-BR-3 was purchased from ATCC and cultured in McCoy's 5a Medium (ATCC modification) supplemented with 10% fetal bovine serum. The SK-BR-3 cell lines were maintained in an atmosphere of 5% CO_2_ in a humidified 37 °C incubator. Cells were plated in 96-well culture plate at 10,000 cells per well in triplicate the day before drug treatment. Cells were treated with antibody or ADCs for 72 h with drug vehicle (PBS, final concentration of 0.05%) and the dilution kept constant across all drug concentrations and controls. Three-fold serial dilution for eight points of antibody and ADCs (ADC 1 66.7 nM to 0.03 nM, ADC 2 66.7 nM to 0.03 nM, Trastuzumab 66.7 nM to 0.03 nM) were used. Cell viability was assessed by Cell Titer-Glo kit (Promega) according to the manufacturer's instruction. At the end of the incubation, luminescence was measured using a SpectraMax i3x Multi Mode Detection Platform (Molecular Devices). Percentage of inhibition, [1 − (RLU value in treated well)/(Average RLU value in untreated control well)] × 100, was calculated based on the measured RLU values. IC50 values were calculated by fitting viability data with a four-parameter logistic equation using GraphPad prism 8.0 software. Data is presented as a mean ± standard error of the mean (SEM) from ≥ 2 independent experiments.

## Supplementary Information

Supplementary Information.
