# Supplementary material for: Selective and predicable amine conjugation sites by kinetic characterization under excess reagents
Source: Sci Rep. 2021 Oct 27;11:21222. doi: 10.1038/s41598-021-00743-3 (PMC8551328; doi:10.1038/s41598-021-00743-3)
Supplement: Supplementary file 1 — Supplementary Information. [file 41598_2021_743_MOESM1_ESM.pdf]

Supporting Information

Selective and Predicable Amine Conjugation Sites by Kinetic Characterization Under Excess Reagents

Table of Content

Figure S1. Effect of reducing agent on the yield of Her-IgG (5.73 μM) conjugated with 2-(2-(2-(2-Azidoethoxy)ethoxy)ethoxy)acetaldehyde (AD-PEG-N_3) linker (3.3 mM). The dashed line indicated the reducing concentrations used for this study.

Figure S2. Far UV spectra of Her-IgG in 50mM Tris-HCl without (blue) and with (red) 0.16 M NaBH3CN

Figure S3. Heat map of Her-IgG. Conjugation level of each amine sites obtained by reaction of 5.73 μM Her-IgG with 0.29 mM, 0.57 mM, 2.58 mM, 5.73 mM, 11.46 mM, 22.92 mM, and 45.84 mM formaldehyde. The higher affinity for formaldehyde was also evidenced by two conjugated formaldehyde molecules per site (dimethyl labeling) versus only one PEG linker per site.

Table S1. Bottom-up proteomics. (a) AA sequence of Her-IgG and (b) identified sequences and conjugation sites of AD-PEG-N_3 (AD) and NHS-PEG-N_3 (NHS) on Her-IgG by chymotrypsin digestion

Supplementary Method. Experiment for payload linker synthesis and conjugation to Her-IgG-PEG-AD

Figure S1. Effect of reducing agent on the yield of Her-IgG (5.73 μM) conjugated with 2-(2-(2-(2-Azidoethoxy)ethoxy)ethoxy)acetaldehyde (AD-PEG-N_3) linker (3.3 mM). The dashed line indicated the reducing concentrations used for this study.

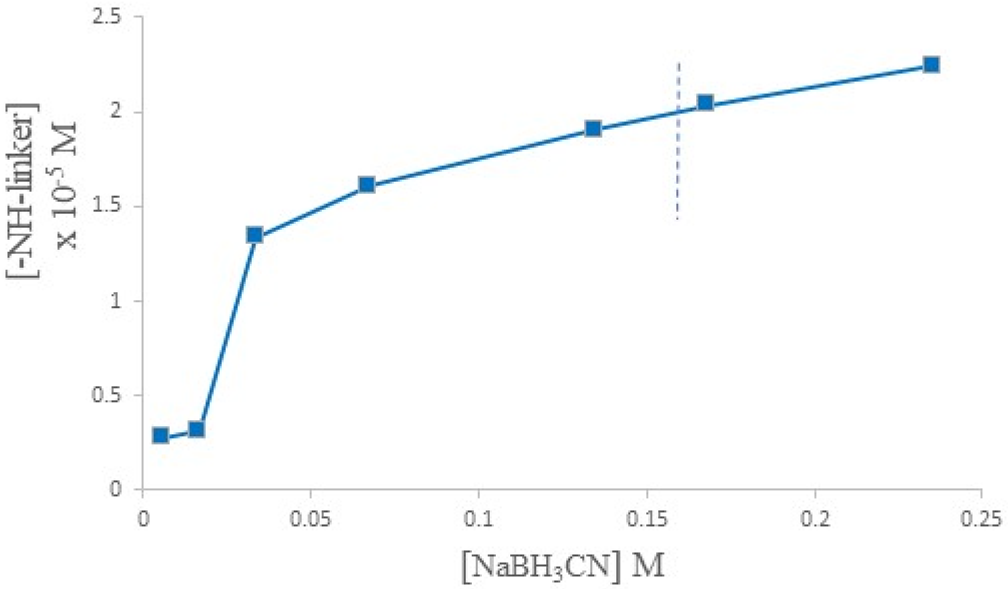

Figure S2. Far UV spectra of Her-IgG in 50mM Tris-HCl without (blue) and with (red) 0.16 M $NaBH_3CN$

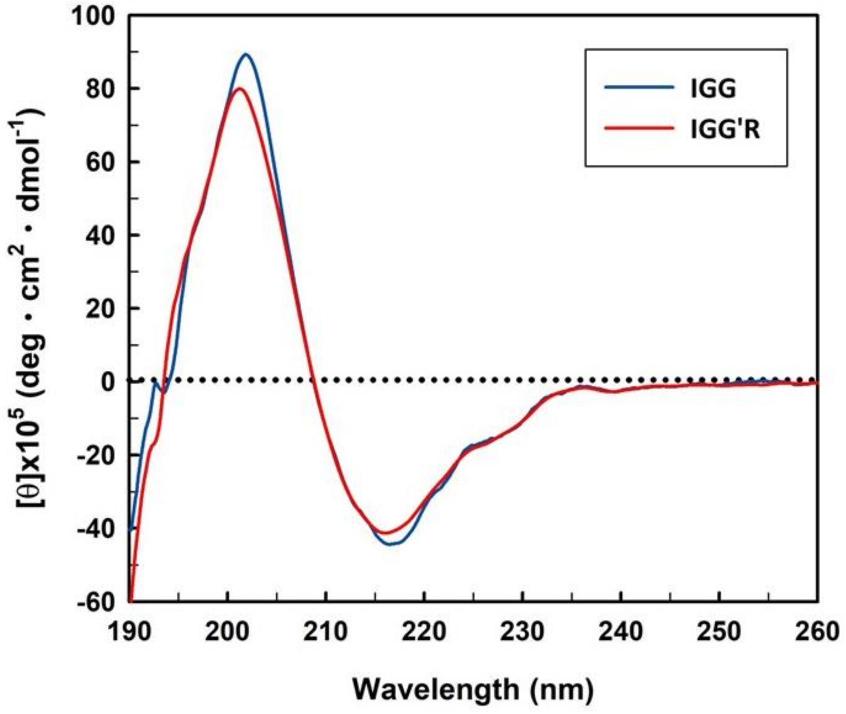

Figure S3. Heat map of Her-IgG. Conjugation level of each amine sites obtained by reaction of 5.73 μM Her-IgG with 0.29 mM, 0.57 mM, 2.58 mM, 5.73 mM, 11.46 mM, 22.92 mM, and 45.84 mM formaldehyde.

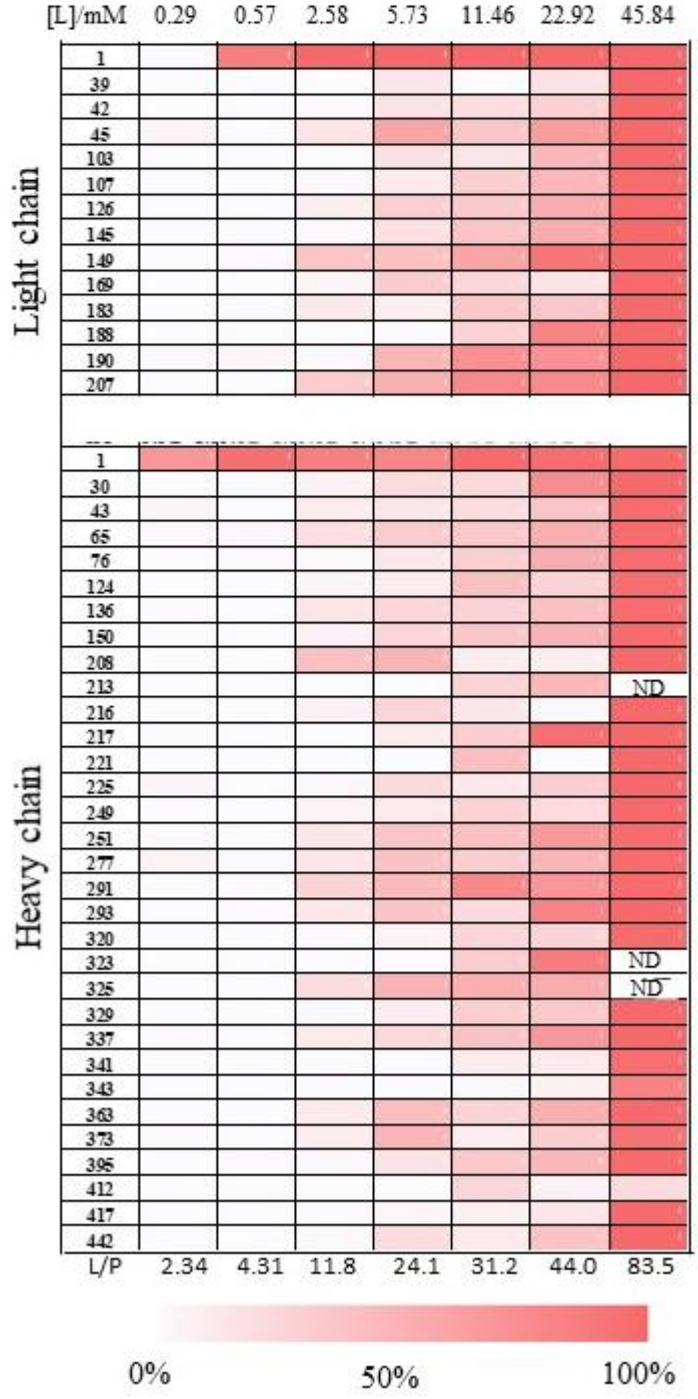

Table S1. Bottom-up proteomics. (a) AA sequence of Her-IgG and (b) identified sequences and conjugation sites of AD-PEG-N_3 (AD) and NHS-PEG-N_3 (NHS) on Her-IgG by chymotrypsin digestion

a.

Light chain

```
1   DIQMTQSPSS LSASVGDRVT ITCRASQDVN TAVAWYQQKP
41  GKAPKLLIYS ASFLYSGVPS RFSGSRSGTD FTLTISSLQP
81  EDFATYYCQQ HYTTPPTFGQ GTKVEIKRTV AAPSVFIFPP
121 SDEQLKSGTA SVVCLLNNFY PREAKVQWKV DNALQSGNSQ
161 ESVTEQDSKD STYSLSSTLT LSKADYEKHK VYACEVTHQG
201 LSSPVTKSFN RGEC
```

Heavy chain

```
1   EVQLVESGGG LVQPGGSLRL SCAASGFNIK DTYIHWVRQA
41  PGKGLEWVAR IYPTNGYTRY ADSVKGRFTI SADTSKNTAY
81  LQMNSLRAED TAVYYCSRWG GDGFYAMDYW GQGTLVTVSS
121 ASTKGPSVFP LAPSSKSTSG GTAALGCLVK DYFPEPVTVS
161 WNSGALTSGV HTFPAVLQSS GLYSLSSVVT VPSSSLGTQT
201 YICNVNHKPS NTKVDKKVEP KSCDKTHTCP PCPAPELLGG
241 PSVFLFPPKP KDTLMISRTP EVTCVVVDVS HEDPEVKFNW
281 YVDGVEVHNA KTKPREEQYN STYRVVSVLT VLHQDWLNGK
321 EYKCKVSNKA LPAPIEKTIS KAKGQPREPQ VYTLPPSREE
361 MTKNQVSLTC LVKGFYPSDI AVEWESNGQP ENNYKTTPPV
401 LDSDGSFFLY SKLTVDKSRW QQGNVFSCSV MHEALHNHYT
441 QKSLSLSPGK
```

b.

light chain_chymotrypsin										
Start	End	Peptide	PTM site	RT	-10lgP	MWexp(Da)	MWthe(Da)	Error(Da)	m/z	z
1	9	DIQMTQSPS	free	24.53	73.06	1005.4432	1005.4437	-0.0005	503.7289	2
			D1:AD(1000.00)	35.39	60.2	1206.5552	1206.5551	1E-04	604.2849	2
1	11	DIQMTQSPSSL	free	31.75	88.34	1205.56	1205.5598	0.0002	603.7873	2
			D1:AD(1000.00)	43.61	63.2	1406.6736	1406.6711	0.0025	704.3441	2
36	45	YQQKPGKAPK	free	2.56	54.29	1143.6406	1143.64	0.0006	572.8276	2
36	47	YQQKPGKAPKLL	free	23.15	81.55	1369.8068	1369.8081	-0.0013	685.9107	2
36	49	YQQKPGKAPKLLIY	free	29.14	38.94	1645.9548	1645.9555	-0.0007	549.6589	3
37	47	QQKPGKAPKLL	free	21.81	53.59	1206.7446	1206.7448	-0.0002	403.2555	3
37	49	QQKPGKAPKLLIY	free	28.49	66.98	1482.8926	1482.8922	0.0004	742.4536	2
93	106	TTPPTFGQGTKVEI	free	32.72	77.37	1474.768	1474.7668	0.0012	738.3913	2
93	107	TTPPTFGQGTKVEIK	free	27.64	80.87	1602.864	1602.8617	0.0023	802.4393	2
99	107	GQGTKVEIK	free	15.08	46.79	958.5438	958.5447	-0.0009	480.2792	2
99	108	GQGTKVEIKR	free	12.46	59.47	1114.6466	1114.6458	0.0008	558.3306	2
103	116	KVEIKRTVAAPSVF	free	28.79	25.04	1543.9056	1543.9086	-0.003	515.6425	3
104	116	VEIKRTVAAPSVF	free	30.78	54.5	1415.8134	1415.8136	-0.0002	708.914	2
105	116	EIKRTVAAPSVF	free	29.52	53.8	1316.7448	1316.7452	-0.0004	659.3797	2
117	130	IFPPSDEQLKSGTA	free	28.51	64.11	1488.7468	1488.746	0.0008	745.3807	2
117	133	IFPPSDEQLKSGTASVV	free	41.73	48.09	1773.9174	1773.9149	0.0025	887.966	2
			K126:AD(1000.00)	36.83	33.59	1975.0378	1975.0262	0.0116	988.5262	2
117	134	IFPPSDEQLKSGTASVVC(+57.02)	free	32.1	63.1	1933.944	1933.9455	-0.0015	645.6553	3
117	135	IFPPSDEQLKSGTASVVC(+57.02)L	free	36.91	76.1	2047.0296	2047.0296	0	683.3505	3
117	136	IFPPSDEQLKSGTASVVC(+57.02)LL	free	41.39	66.39	2160.1194	2160.1137	0.0057	1081.067	2
126	133	KSGTASVV	free	15.85	26.58	747.4126	747.4127	-1E-04	374.7136	2
			K126:AD(1000.00)	26.88	34.94	948.524	948.524	0	475.2693	2
126	134	KSGTASVVC	free	15.65	57.42	907.4432	907.4433	-1E-04	454.7289	2
			K126:AD(1000.00)	25.64	52.59	1108.5548	1108.5547	0.0001	555.2847	2
126	135	TKGPSVFPL	free	28.9	55.45	1020.527	1020.5274	-0.0004	511.2708	2
126	136	KSGTASVVC(+57.02)LL	free	35.56	54.37	1133.6112	1133.6115	-0.0003	567.8129	2
			K126:AD(1000.00)	39.29	40.65	1334.7234	1334.7228	0.0006	668.369	2
136	145	LN(+.98)NFYPREAK	free	24.78	33.18	1251.6234	1251.6247	-0.0013	418.2151	3
136	145	LNNFYPREAK	free	23.72	82.18	1250.6408	1250.6407	0.0001	626.3277	2
137	145	N(+.98)NFYPREAK	free	22.62	67.33	1138.5398	1138.5407	-0.0009	570.2772	2
137	148	NNFYPREAKVQW	free	31.03	96.05	1550.7638	1550.763	0.0008	776.3892	2
			K145:AD(1000.00)	34.52	36.94	1751.8734	1751.8743	-0.0009	584.9651	3
138	148	NFYPREAKVQW	free	31.09	55.07	1436.7196	1436.7201	-0.0005	719.3671	2
140	148	YPREAKVQW	free	26.63	27.21	1175.6085	1175.6087	-0.0002	392.8768	3
143	148	EAKVQW	free	25.95	23.44	759.3904	759.3915	-0.0011	380.7025	2
149	155	KVDNALQ	free	15.01	45.18	786.4234	786.4236	-0.0002	394.219	2
149	170	KVDNALQSGNSQESVTEQDSKD	free	21.5	100.91	2378.0892	2378.0833	0.0059	793.7037	3
153	173	ALQSGNSQESVTEQDSKDSTY	free	23.24	112.72	2272.9954	2272.9931	0.0023	1137.505	2
155	173	QSGNSQESVTEQDSKDSTY	free	20.96	116.15	2088.8774	2088.872	0.0054	1045.446	2
156	173	SGNSQESVTEQDSKDSTY	free	20.83	101.96	1960.8188	1960.8134	0.0054	981.4167	2
			K169:AD(1000.00)	26.8	99.17	2161.9294	2161.9247	0.0047	1081.972	2
159	173	SQESVTEQDSKDSTY	free	20.74	56.21	1702.723	1702.717	0.006	852.3688	2
180	186	TLSKADY	free	19.18	47.16	796.3958	796.3967	-0.0009	399.2052	2
			K183:AD(1000.00)	28.14	53.05	997.5058	997.508	-0.0022	499.7602	2
180	189	TLSKADYEKH	free	11.67	34.13	1190.5929	1190.5931	-0.0002	397.8716	3
			K183:AD(39.44)	22.81	18.2	1391.7038	1391.7045	-0.0007	696.8592	2
180	192	TLSKADYEKHKVY	free	20.09	103.99	1580.8238	1580.8198	0.004	791.4192	2
			K183:AD(47.70)	24.34	82.78	1781.9346	1781.9312	0.0034	891.9746	2
182	192	SKADYEKHKVY	free	16.57	36.29	1366.686	1366.6881	-0.0021	456.5693	3
			K183:AD(16.28)	22.17	22.21	1567.7985	1567.7994	-0.0009	523.6068	3
193	207	AC(+57.02)EVTHQGLSSPVTK	free	22.78	113.55	1612.7886	1612.7879	0.0007	807.4016	2
193	209	AC(+57.02)EVTHQGLSSPVTKSF	free	28.37	123.5	1846.8916	1846.8884	0.0032	924.4531	2
193	214	AC(+57.02)EVTHQGLSSPVTKSFNRGEC(+57.02)	free	25.91	118.66	2463.1254	2463.1271	-0.0017	822.0491	3
199	209	QGLSSPVTKSF	free	29.56	91.14	1149.603	1149.603	0	575.8088	2
			K207:AD(1000.00)	35.21	42.98	1350.7146	1350.7143	0.0003	676.3646	2
200	209	GLSSPVTKSF	free	29.2	77.34	1021.5438	1021.5444	-0.0006	511.7792	2
202	207	SSPVTK	free	10.07	30.62	617.3388	617.3384	0.0004	309.6767	2
202	209	SSPVTKSF	free	23.06	64.4	851.439	851.4389	0.0001	426.7268	2
			K207:AD(1000.00)	31.5	60.93	1052.5518	1052.5502	0.0016	527.2832	2
202	214	SSPVTKSFN(+.98)RGEC(+57.02)	free	20.91	87.36	1468.6634	1468.6616	0.0018	735.339	2

138	148	NFYPREAKVQW	free	31.09	55.07	1436.7196	1436.7201	-0.0005	719.3671	2
140	148	YPREAKVQW	free	26.63	27.21	1175.6085	1175.6087	-0.0002	392.8768	3
143	148	EAKVQW	free	25.95	23.44	759.3904	759.3915	-0.0011	380.7025	2
149	155	KVDNALQ	free	15.01	45.18	786.4234	786.4236	-0.0002	394.219	2
149	170	KVDNALQSGNSQESVTEQDSKD	free	21.5	100.91	2378.0892	2378.0833	0.0059	793.7037	3
153	173	ALQSGNSQESVTEQDSKDSTY	free	23.24	112.72	2272.9954	2272.9931	0.0023	1137.505	2
155	173	QSGNSQESVTEQDSKDSTY	free	20.96	116.15	2088.8774	2088.872	0.0054	1045.446	2
156	173	SGNSQESVTEQDSKDSTY	free	20.83	101.96	1960.8188	1960.8134	0.0054	981.4167	2
			K169:AD(1000.00)	26.8	99.17	2161.9294	2161.9247	0.0047	1081.972	2
159	173	SQESVTEQDSKDSTY	free	20.74	56.21	1702.723	1702.717	0.006	852.3688	2
180	186	TLSKADY	free	19.18	47.16	796.3958	796.3967	-0.0009	399.2052	2
			K183:AD(1000.00)	28.14	53.05	997.5058	997.508	-0.0022	499.7602	2
180	189	TLSKADYEKH	free	11.67	34.13	1190.5929	1190.5931	-0.0002	397.8716	3
			K183:AD(39.44)	22.81	18.2	1391.7038	1391.7045	-0.0007	696.8592	2
180	192	TLSKADYEKHKVY	free	20.09	103.99	1580.8238	1580.8198	0.004	791.4192	2
			K183:AD(47.70)	24.34	82.78	1781.9346	1781.9312	0.0034	891.9746	2
182	192	SKADYEKHKVY	free	16.57	36.29	1366.686	1366.6881	-0.0021	456.5693	3
			K183:AD(16.28)	22.17	22.21	1567.7985	1567.7994	-0.0009	523.6068	3
193	207	AC(+57.02)EVTHQGLSSPVTK	free	22.78	113.55	1612.7886	1612.7879	0.0007	807.4016	2
193	209	AC(+57.02)EVTHQGLSSPVTKSF	free	28.37	123.5	1846.8916	1846.8884	0.0032	924.4531	2
193	214	AC(+57.02)EVTHQGLSSPVTKSFNRGEC(+57.02)	free	25.91	118.66	2463.1254	2463.1271	-0.0017	822.0491	3
199	209	QGLSSPVTKSF	free	29.56	91.14	1149.603	1149.603	0	575.8088	2
			K207:AD(1000.00)	35.21	42.98	1350.7146	1350.7143	0.0003	676.3646	2
200	209	GLSSPVTKSF	free	29.2	77.34	1021.5438	1021.5444	-0.0006	511.7792	2
202	207	SSPVTK	free	10.07	30.62	617.3388	617.3384	0.0004	309.6767	2
202	209	SSPVTKSF	free	23.06	64.4	851.439	851.4389	0.0001	426.7268	2
			K207:AD(1000.00)	31.5	60.93	1052.5518	1052.5502	0.0016	527.2832	2
202	214	SSPVTKSFN(+.98)RGEC(+57.02)	free	20.91	87.36	1468.6634	1468.6616	0.0018	735.339	2
Heavy chain_chymptrysin										
Start	End	Peptide	PTM site	RT	-10lgP	MWexp(Da)	MWthe(Da)	Error(Da)	m/z	z
1	11	EVQLVESGGGL	E1:Pyro-glu from E(1000.00)	40.53	78.31	1068.5448	1068.5357	0.0091	535.2797	2
			free	33.47	90.18	1086.5548	1086.5557	-0.0009	544.2847	2
			E1:AD(1000.00)	40.75	70.16	1287.6676	1287.6671	0.0005	644.8411	2
1	18	EVQLVESGGGLVQPGGSL	E1:Pyro-glu from E(1000.00)	37.42	90.95	1706.886	1706.884	0.002	854.4503	2
			free	49.67	99.54	1724.897	1724.8945	0.0025	863.4558	2
			E1:AD(1000.00)	42.64	49.8	1926.0106	1926.0058	0.0048	964.0126	2

1	20	EVQLVESGGGLVQPGGSLRL	free	38.62	127.12	1994.084	1994.0797	0.0043	998.0493	2
			E1:AD(1000.00)	42.68	101.53	2195.1906	2195.191	-0.0004	732.7375	3
21	33	SC(+57.02)AASGFNIKDTY	free	35.63	32.63	1432.622	1432.6293	-0.0073	717.3183	2
26	33	GFNIKDTY	free	29.43	66.53	956.4586	956.4603	-0.0017	479.2366	2
28	33	NIKDTY	N28:Deamidation (NQ)(1000.00)	22.6	37.07	753.355	753.3545	0.0005	377.6848	2
			free	18.72	37.6	752.3706	752.3705	0.0001	377.1926	2
			K30:AD(1000.00)	27.9	38.84	953.4788	953.4818	-0.003	477.7467	2
28	36	NIKDTYIHW	free	32.22	87.26	1188.5961	1188.5927	0.0034	397.206	3
			K30:AD(1000.00)	36.67	21.09	1389.7035	1389.7041	-0.0006	464.2418	3
29	33	IKDTY	free	16.3	18.74	638.3278	638.3275	0.0003	320.1712	2
37	47	VRQAPGKGLEW	free	27.23	40.33	1239.6726	1239.6724	0.0002	620.8436	2
			K43:AD(1000.00)	33.31	91.21	1440.7816	1440.7837	-0.0021	721.3981	2
39	47	QAPGKGLEW	free	30.1	80.96	984.5026	984.5029	-0.0003	493.2586	2
43	47	KGLEW	free	29.68	27.61	631.3328	631.3329	-1E-04	316.6737	2
58	68	TRYADSVKGRF	free	22.3	90.61	1298.675	1298.6731	0.0019	650.3448	2
60	68	YADSVKGRF	free	22.51	22.12	1041.524	1041.5243	-0.0003	521.7693	2
61	68	ADSVKGRF	free	18.08	64.99	878.4606	878.461	-0.0004	440.2376	2
			K65:AD(1000.00)	26.66	29.06	1079.5737	1079.5724	0.0013	360.8652	3
69	80	TISADTSKNTAY	N77:Deamidation (NQ)(1000.00)	22.01	58.34	1271.5982	1271.5881	0.0101	636.8064	2
			free	21.7	58.34	1270.605	1270.6041	0.0009	636.3098	2
			K76:AD(1000.00)	28.02	84.02	1471.7124	1471.7154	-0.003	736.8635	2
69	81	TISADTSKNTAYL	free	27.89	89.73	1383.6874	1383.6882	-0.0008	692.851	2
			K76:AD(1000.00)	33.41	58.33	1584.8008	1584.7995	0.0013	793.4077	2
69	84	TISADTSKNTAYLQMN	free	29.05	72.14	1756.834	1756.8301	0.0039	879.4243	2
70	80	ISADTSKNTAY	free	20.26	84.8	1169.556	1169.5564	-0.0004	585.7853	2
71	80	SADTSKNTAY	free	11.34	50.36	1056.4722	1056.4724	-0.0002	529.2434	2
111	124	GQGTLVTVSSASTK	free	24.67	115.56	1334.7044	1334.7042	0.0002	668.3595	2
116	127	VTVSSASTKGPS	free	18.71	68.21	1119.5772	1119.5772	0	560.7959	2
116	129	VTVSSASTKGPSVF	free	29.71	103.5	1365.7136	1365.714	-0.0004	683.8641	2
116	131	VTVSSASTKGPSVFPL	free	36.58	107.11	1575.8504	1575.8508	-0.0004	788.9325	2
116	136	VTVSSASTKGPSVFPLAPSSK	free	31.83	117.85	2046.099	2046.0997	-0.0007	683.0403	3
116	145	VTVSSASTKGPSVFPLAPSSKSTSGGTAAL	free	34.2	78.76	2791.467	2791.4604	0.0066	931.4963	3
117	131	TVSSASTKGPSVFPL	free	35.82	99.24	1476.7826	1476.7824	0.0002	739.3986	2
118	131	VSSASTKGPSVFPL	free	35.63	116.45	1375.734	1375.7347	-0.0007	688.8743	2
			K124:AD(1000.00)	36.65	30.46	1576.8532	1576.8461	0.0071	789.4339	2
118	134	VSSASTKGPSVFPLAPS	free	34.55	81.1	1630.8582	1630.8566	0.0016	816.4364	2
119	129	SSASTKGPSVF	free	26.43	89.46	1066.5284	1066.5295	-0.0011	534.2715	2
119	131	SSASTKGPSVFPL	free	35.29	78.85	1276.6664	1276.6663	1E-04	639.3405	2
119	134	SSASTKGPSVFPLAPS	free	34.27	86.97	1531.7898	1531.7882	0.0016	766.9022	2
120	129	SASTKGPSVF	free	26.32	86.69	979.4964	979.4975	-0.0011	490.7555	2
120	131	SASTKGPSVFPL	free	35.32	82.89	1189.635	1189.6343	0.0007	595.8248	2
			K124:AD(1000.00)	40	42.08	1390.748	1390.7456	0.0024	696.3813	2
120	134	SASTKGPSVFPLAPS	free	34.35	16.7	1444.7572	1444.7562	0.001	723.3859	2
120	136	SASTKGPSVFPLAPSSK	free	30.24	119.28	1659.8854	1659.8832	0.0022	830.95	2
120	145	SASTKGPSVFPLAPSSKSTSGGTAAL	free	33.21	96.08	2405.2467	2405.2438	0.0029	802.7562	3
122	129	STKGPSVF	free	25.88	60.27	821.4276	821.4283	-0.0007	411.7211	2
122	131	STKGPSVFPL	free	35.46	70.73	1031.5646	1031.5652	-0.0006	516.7896	2
122	134	STKGPSVFPLAPS	free	34.49	72.9	1286.6874	1286.6871	0.0003	644.351	2
122	145	STKGPSVFPLAPSSKSTSGGTAAL	free	33.21	96.08	2247.1743	2247.1747	-0.0004	750.0654	3
123	134	TKGPSVFPLAPS	free	34.66	74.57	1199.655	1199.655	0	600.8348	2
123	136	TKGPSVFPLAPSSK	free	30.3	98.37	1414.7822	1414.782	0.0002	708.3984	2
124	131	KGPSVFPL	free	35.44	67.75	843.4856	843.4854	0.0002	422.7501	2
			K124:AD(1000.00)	40.14	30.44	1044.5962	1044.5968	-0.0006	523.3054	2
124	134	KGPSVFPLAPS	free	34.63	59.98	1098.6062	1098.6073	-0.0011	550.3104	2
124	138	KGPSVFPLAPSSKST	free	30.24	79.11	1501.8072	1501.814	-0.0068	751.9109	2
124	145	KGPSVFPLAPSSKSTSGGTAAL	free	33.17	134.21	2059.0974	2059.095	0.0024	1030.556	2
116	136	VTVSSASTKGPSVFPLAPSSK	free	31.83	117.85	2046.099	2046.0997	-0.0007	683.0403	3
116	145	VTVSSASTKGPSVFPLAPSSKSTSGGTAAL	free	34.2	78.76	2791.467	2791.4604	0.0066	931.4963	3
120	136	SASTKGPSVFPLAPSSK	free	30.24	119.28	1659.8854	1659.8832	0.0022	830.95	2
120	145	SASTKGPSVFPLAPSSKSTSGGTAAL	free	33.21	79.11	2405.2467	2405.2438	0.0029	802.7562	3
122	145	STKGPSVFPLAPSSKSTSGGTAAL	free	33.21	96.08	2247.1743	2247.1747	-0.0004	750.0654	3
125	136	GPSVFPLAPSSK	free	33.14	73.75	1185.6422	1185.6394	0.0028	593.8284	2
124	138	KGPSVFPLAPSSKST	free	30.24	79.11	1501.8072	1501.814	-0.0068	751.9109	2
124	145	KGPSVFPLAPSSKSTSGGTAAL	free	33.17	134.21	2059.0974	2059.095	0.0024	1030.556	2
125	138	GPSVFPLAPSSKST	free	32.93	82.75	1373.7164	1373.7191	-0.0027	687.8655	2
125	145	GPSVFPLAPSSKSTSGGTAAL	free	36.2	89.13	1931	1931	0	966.5073	2
128	138	VFPLAPSSKST	free	29.64	64.59	1132.6118	1132.6128	-0.001	567.3132	2
128	145	VFPLAPSSKSTSGGTAAL	free	33.99	93.73	1689.894	1689.8938	0.0002	845.9543	2
130	145	PLAPSSKSTSGGTAAL	free	25.36	102.22	1443.7572	1443.7569	0.0003	722.8859	2
132	145	APSSKSTSGGTAAL	free	20.19	90.37	1233.6196	1233.6201	-0.0005	617.8171	2
			K136:AD(1000.00)	27.26	53.72	1434.7334	1434.7314	0.002	718.374	2
134	145	SSKSTSGGTAAL	free	17.93	74.06	1065.5292	1065.5302	-0.001	533.7719	2

135	145	SKSTSGGTAAL	free	17.7	68.56	978.4976	978.4982	-0.0006	490.2561	2
			K136:AD(1000.00)	23.26	30.44	1179.6102	1179.6095	0.0007	590.8124	2
146	152	GC(+57.02)LVKDY	free	24.21	53.36	853.4034	853.4004	0.003	427.709	2
146	157	GC(+57.02)LVKDYFPEPV	free	36.64	60.38	1422.6914	1422.6853	0.0061	712.353	2
146	159	GC(+57.02)LVKDYFPEPVTV	free	38.27	88.46	1622.8024	1622.8015	0.0009	812.4085	2
			K150:AD(1000.00)	41.72	21.44	1823.9066	1823.9128	-0.0062	912.9606	2
146	161	GC(+57.02)LVKDYFPEPVTVSW	free	42.12	112.97	1895.9144	1895.9128	0.0016	948.9645	2
149	159	VKDYFPEPVTV	free	35.21	94.52	1292.665	1292.6653	-0.0003	647.3398	2
149	161	VKDYFPEPVTVSW	free	40.13	57.41	1565.781	1565.7766	0.0044	783.8978	2
202	212	IC(+57.02)NVNHKPSNT	free	10.67	57.24	1282.6098	1283.5928	-0.983	642.3122	2
			K208:AD(1000.00)	21.69	41.75	1483.7206	1483.7202	0.0004	742.8676	2
207	212	HKPSNT	free	2.46	37.6	682.3402	682.3398	0.0004	342.1774	2
213	221	KVDKKVEPK	free	2.43	43.06	1069.65	1069.6495	0.0005	357.5573	3
213	227	KVDKKVEPKSC(+57.02)DKTH	free	8.73	87.33	1797.9411	1797.9407	0.0004	600.321	3
213	238	KVDKKVEPKSC(+57.02)DKTHTC(+57.02)PPC(+57.02)PAPELL	free	25.66	57.16	3033.5151	3033.5086	0.0065	1012.179	3
213	227	KVDKKVEPKSC(+57.02)DKTH	free	8.73	87.33	1797.9411	1797.9407	0.0004	600.321	3
213	238	KVDKKVEPKSC(+57.02)DKTHTC(+57.02)PPC(+57.02)PAPELL	free	25.66	57.16	3033.5151	3033.5086	0.0065	1012.179	3
222	227	SC(+57.02)DKTH	free	2.54	75.71	746.3016	746.3017	-1E-04	374.1581	2
222	238	SC(+57.02)DK(+201.11)THTC(+57.02)PPC(+57.02)PAPELL	free	33.83	45.61	2182.9821	2182.981	0.0011	728.668	3
222	238	SC(+57.02)DKTHTC(+57.02)PPC(+57.02)PAPELL	free	30.74	80.06	1981.8766	1981.8696	0.007	991.9456	2
222	244	SC(+57.02)DKTHTC(+57.02)PPC(+57.02)PAPELLGGPSVF	free	37.45	112.41	2526.1394	2526.1342	0.0052	1264.077	2
224	238	DKTHTC(+57.02)PPC(+57.02)PAPELL	free	31.6	112.85	1734.8074	1734.8069	0.0005	868.411	2
224	244	DKTHTC(+57.02)PPC(+57.02)PAPELLGGPSVF	free	38.29	97.32	2279.0734	2279.0715	0.0019	1140.544	2
245	254	LFPPKPKDTL	free	28.12	25.97	1154.6697	1154.6699	-0.0002	385.8972	3
245	255	LFPPKPKDTLM	free	30.16	54.09	1285.7118	1285.7104	0.0014	643.8632	2
245	254	LFPPKPKDTL	free	28.12	25.97	1154.6697	1154.6699	-0.0002	385.8972	3
245	255	LFPPKPKDTLM	free	30.16	54.09	1285.7118	1285.7104	0.0014	643.8632	2
255	278	MISRTPEVTC(+57.02)VVVDVSHEDPEVKF	free	36.74	64.33	2772.3452	2772.3463	-0.0011	694.0936	4
256	278	ISRTPEVTC(+57.02)VVVDVSHEDPEVKF	free	35.95	111.88	2641.3114	2641.3058	0.0056	1321.663	2
264	278	C(+57.02)VVVDVSHEDPEVKF	free	32.82	115.13	1757.8338	1757.8295	0.0043	879.9242	2
265	278	VVVDVSHEDPEVKF	free	31.91	123.95	1597.8004	1597.7988	0.0016	799.9075	2
266	278	VVDVSHEDPEVKF	free	29.84	29.95	1498.7283	1498.7304	-0.0021	500.5834	3
282	299	VDGVEVHN(+.98)AKTKPREEQY	free	21.53	90.27	2099.0271	2099.0283	-0.0012	700.683	3
282	299	VDGVEVHNAKTKPREEQY	free	19.41	93.12	2098.0458	2098.0443	0.0015	700.3559	3
282	303	VDGVEVHNAKTKPREEQYN(+.98)STY	free	21.62	74.17	2564.2161	2564.2143	0.0018	855.746	3
289	299	NAKTKPREEQY	free	9.02	70.3	1362.6902	1362.6891	0.0011	682.3524	2

290	303	AKTKPREEQYN(+.98)STY	free	17.29	56.89	1714.8194	1714.8162	0.0032	858.417	2
289	303	NAKTKPREEQYNSTY	free	17.62	21.56	1828.857	1829.8431	-0.9861	610.6263	3
			K291:AD(33.18)	22.36	45.77	2029.9794	2029.9704	0.009	1015.997	2
282	299	VDGVEVHN(+.98)AKTKPREEQY	free	21.53	90.27	2099.0271	2099.0283	-0.0012	700.683	3
282	299	VDGVEVHNAKTKPREEQY	free	19.41	93.12	2098.0458	2098.0443	0.0015	700.3559	3
282	303	VDGVEVHNAKTKPREEQYN(+.98)STY	free	21.62	74.17	2564.2161	2564.2143	0.0018	855.746	3
289	299	NAKTKPREEQY	free	9.02	70.3	1362.6902	1362.6891	0.0011	682.3524	2
289	303	NAKTKPREEQYN(+.98)STY	free	17.62	21.56	1828.857	1828.8591	-0.0021	610.6263	3
			K293:AD(33.18)	22.36	45.77	2029.9794	2029.9704	0.009	1015.997	2
290	303	AKTKPREEQYN(+.98)STY	free	17.29	56.89	1714.8194	1714.8162	0.0032	858.417	2
292	303	TKPREEQYN(+.98)STY	free	19.32	70	1515.6866	1515.6841	0.0025	758.8506	2
			K293:AD(1000.00)	24.71	34.96	1716.7978	1716.7955	0.0023	859.4062	2
310	322	TVLHQDWLN(+.98)GKEY	free, N318:Deamidation (NQ)(97.65)	42.23	102.89	1602.7726	1602.7678	0.0048	802.3936	2
			free	31.38	111.73	1601.7878	1601.7838	0.004	801.9012	2
			K320:AD(1000.00)	35.82	101.56	1803.8812	1803.8792	0.002	902.9479	2
311	322	VLHQDWLN(+.98)GKEY	free	30.4	83.85	1501.7206	1501.7201	0.0005	751.8676	2
313	322	HQDWLNGKEY	free	33.47	63.13	1288.5754	1288.5836	-0.0082	645.295	2
			N318:Deamidation (NQ):127.66	27.25	99.33	1289.568	1289.5676	0.0004	645.7913	2
317	322	LNGKEY	free	13.4	29.72	722.3602	722.3599	0.0003	362.1874	2
			N318:Deamidation (NQ):1000.00)	17.53	36.33	723.3444	723.3439	0.0005	362.6795	2
323	328	KC(+57.02)KVSN	free	2.51	54.56	734.3752	734.3745	0.0007	368.1949	2
323	329	KC(+57.02)KVSNK	free	2.38	27.55	862.4692	862.4695	-0.0003	432.2419	2
323	337	KC(+57.02)KVSN(+.98)KALPAPIEK	N328:Deamidation (NQ):1000.00)	22.05	17.98	1682.9384	1682.9389	-0.0005	421.7419	4
323	337	KC(+57.02)KVSNKALPAPIEK	free	21.05	49.62	1681.9536	1681.9549	-0.0013	561.6585	3
326	337	VSNKALPAPIEK	free	23.86	97.88	1265.7344	1265.7343	1E-04	633.8745	2
328	339	NKALPAPIEKTI	free	28.49	80.22	1293.7646	1293.7656	-0.001	647.8896	2
328	340	NKALPAPIEKTIS	free	27.5	69.69	1380.7966	1380.7976	-0.001	691.4056	2
329	337	KALPAPIEK	free	22.04	73.87	965.5906	965.5909	-0.0003	483.8026	2
			K329:AD(68.03)	28.71	52.85	1166.7008	1166.7023	-0.0015	584.3577	2
329	339	KALPAPIEKTI	free	28.51	52.83	1179.7218	1179.7227	-0.0009	590.8682	2
329	340	KALPAPIEKTIS	free	28.3	84.98	1266.7545	1266.7547	-0.0002	423.2588	3
329	341	KALPAPIEKTISK	free	24.6	86.42	1394.8512	1394.8497	0.0015	698.4329	2
330	337	ALPAPIEK	free	25.35	26.36	837.4958	837.496	-0.0002	419.7552	2
330	338	ALPAPIEKT	free	26.3	53.37	938.5422	938.5437	-0.0015	470.2784	2
330	339	ALPAPIEKTI	free	32.2	80.74	1051.6294	1051.6277	0.0017	526.822	2

330	342	ALPAPIEKTISKA	free	30.48	86.51	1337.7916	1337.7918	-0.0002	669.9031	2
332	337	PAPIEK	free	25.41	45.27	653.3752	653.3748	0.0004	327.6949	2
332	338	PAPIEKT	free	26.38	51.67	754.423	754.4225	0.0005	378.2188	2
338	343	TISKAK	free	2.58	31.94	646.4018	646.4014	0.0004	324.2082	2
338	350	TISKAKGQPREPQ	free	9.32	17.37	1438.788	1438.7892	-0.0012	480.6033	3
338	352	TISKAKGQPREPQVY	free	20.5	108.22	1700.9244	1700.9209	0.0035	851.4695	2
			K343:AD(8.22)	24.65	47.5	1902.0318	1902.0323	-0.0005	635.0179	3
339	352	ISKAKGQPREPQVY	free	19.94	96.09	1599.875	1599.8733	0.0017	800.9448	2
340	352	SKAKGQPREPQVY	free	18.71	89.82	1486.7912	1486.7892	0.002	744.4029	2
341	352	KAKGQPREPQVY	free	18.42	82.7	1399.758	1399.7572	0.0008	700.8863	2
342	352	AKGQPREPQVY	free	24.89	53.37	1271.663	1271.6622	0.0008	636.8388	2
			K343:AD(1000.00)	25.58	44.16	1472.7738	1472.7736	0.0002	737.3942	2
343	352	KGQPREPQVY	free	20.55	62.68	1200.625	1200.6251	-1E-04	601.3198	2
			K343:AD(1000.00)	25.26	40.58	1401.7362	1401.7362	0	701.8754	2
353	364	TLPPSREEMTKN	free	20.15	53.64	1401.693	1401.6922	0.0008	701.8538	2
353	368	TLPPSREEMTKNQVSL	free	28.21	84.85	1828.9392	1828.9353	0.0039	915.4769	2
353	369	TLPPSREEMTKNQVSLT	free	28.65	44.96	1929.9864	1929.983	0.0034	966.0005	2
362	368	TKNQVSL	free	22.1	58.17	788.4396	788.4392	0.0004	395.2271	2
			K363:AD(1000.00)	30.01	29.16	989.5496	989.5506	-0.001	495.7821	2
369	375	TC(+57.02)LVKGF	free	29.99	83.57	823.4258	823.4262	-0.0004	412.7202	2
370	375	C(+57.02)LVKGF	free	29.45	69.85	722.3786	722.3785	1E-04	362.1966	2
			K373:AD(1000.00)	35.85	30.98	923.49	923.4899	1E-04	462.7523	2
370	384	C(+57.02)LVKGFYPSDIAVEW	free	39.06	36.16	1782.8708	1782.8651	0.0057	892.4427	2
385	408	ESNGQPENNYKTTPPVLDSDGSFF	N393:Deamidation (NQ):10.03	38.71	69.47	2643.1714	2643.1613	0.0101	1322.593	2
			free	38.33	90.11	2642.1834	2642.1773	0.0061	1322.099	2
395	407	KTTPPVLDSDGSF	free	31.18	120.41	1362.667	1362.6667	0.0003	682.3408	2
395	408	KTTPPVLDSDGSFF	free	37.32	128.74	1509.7378	1509.7352	0.0026	755.8762	2
			K395:AD(1000.00)	40.85	98.29	1710.851	1710.8465	0.0045	856.4328	2
395	410	KTTPPVLDSDGSFFLY	free	41.49	18.86	1785.8862	1785.8825	0.0037	893.9504	2
			K395:AD(1000.00)	44.21	64.88	1986.999	1986.9939	0.0051	994.5068	2
411	426	SKLTVDKSRWQQGNVF	free	29.44	102.39	1891.9946	1891.9904	0.0042	947.0046	2
411	420	SKLTVDKSRW	free	23.28	99.9	1218.6702	1218.6721	-0.0019	407.2307	3
411	426	SKLTVDKSRWQQGNVF	free	29.44	102.39	1891.9946	1891.9904	0.0042	947.0046	2
414	420	TVDKSRW	free	18.04	56.58	890.4598	890.461	-0.0012	446.2372	2
414	426	TVDKSRWQQGNVF	free	28.82	84.88	1563.7808	1563.7794	0.0014	782.8977	2
427	444	SC(+57.02)SVMHEALHN(+.98)HYTQKSL	free	25.7	54.97	2141.9583	2141.9622	-0.0039	714.9934	3
427	444	SC(+57.02)SVMHEALHNHYTQKSL	free	25.21	77.95	2140.9794	2140.9782	0.0012	714.6671	3
440	449	TQKSLSLSPG	free	24.72	74.77	1016.5494	1016.5502	-0.0008	509.282	2
440	444	TQK(+201.11)SL	K442:AD_K(1000.00)	26.27	36.04	776.439	776.435	0.004	389.2268	2

Light Chain_chymotrypsin										
Start	End	Peptide	PTM	RT	-10lgP	MWexp(Da)	MWthe(Da)	Error(Da)	m/z	z
1	9	DIQMTQSPS	free	24.13	66.05	1005.4432	1005.4437	-0.0005	503.7289	2
			D1:NHS(1000.00)	39.5	22.96	1220.5338	1220.5343	-0.0005	611.2742	2
1	11	DIQMTQSPSSL	free	31.11	89.83	1205.5598	1205.5598	0	603.7872	2
			D1:NHS(1000.00)	43.55	26.07	1420.6532	1420.6504	0.0028	711.3339	2
36	47	YQQKPGKAPKLL	free	22.75	38.09	1369.8069	1369.8081	-0.0012	457.6096	3
			K42:NHS(26.52)	32.14	46.33	1584.9008	1584.8987	0.0021	793.4577	2
			K39:NHS(1000.00);K42:NHS(64.77)	41.45	39.34	1799.995	1799.995	0	901.0048	2
37	47	QQKPGKAPKLL	K39:NHS(1000.00);K45:NHS(73.31)	41.14	32.17	1636.9302	1636.926	0.0042	819.4724	2
99	106	GQGTKVEI	free	22.57	51.53	830.45	830.4498	0.0002	416.2323	2
99	107	GQGTKVEIK	free	14.45	17.22	958.5444	958.5447	-0.0003	480.2795	2
99	108	GQGTKVEIK(+215.09)R	K107:NHS(59.24)	26.25	24.62	1329.7358	1329.7365	-0.0007	665.8752	2
			K103:NHS(1000.00);K107:NHS(1000.00)	35.47	32.08	1544.828	1544.828	0	773.4213	2
99	109	GQGTKVEIK(+215.09)RT	K107:NHS(48.12)	26.72	26.12	1430.7831	1430.7841	-0.001	477.935	3
103	108	KVEIK(+215.09)R	K107:NHS(63.97)	25.54	19.7	986.5864	986.5873	-0.0009	494.3005	2
93	108	TTPPTFGQGTK(+215.09)VEIK(+215.09)R	K103:NHS(1000.00);K107:NHS(1000.00)	39.18	78.19	2189.1554	2189.1441	0.0113	1095.585	2
99	107	GQGTKVEIK	free	14.45	17.22	958.5444	958.5447	-0.0003	480.2795	2
107	116	KRTVAAPSVF	free	26.62	44.88	1074.6172	1074.6186	-0.0014	538.3159	2
117	133	IFPPSDEQLKSGTASVV	free	32.26	58.18	1773.9156	1773.9149	0.0007	887.9651	2
117	134	IFPPSDEQLKSGTASVVC(+57.02)	free	31.31	61.85	1933.947	1933.9455	0.0015	967.9808	2
117	135	IFPPSDEQLKSGTASVVC(+57.02)L	free	36.14	99.05	2047.0314	2047.0296	0.0018	1024.523	2
126	134	KSGTASVVC(+57.02)	free	15.16	39.86	907.4424	907.4433	-0.0009	454.7285	2
126	135	KSGTASVVC(+57.02)L	free	28.16	38.2	1020.5274	1020.5274	0	511.271	2
126	136	KSGTASVVC(+57.02)LL	free	34.77	36.69	1133.611	1133.6115	-0.0005	567.8128	2
136	148	LNNFYPREAKVQW	free	32.17	38.44	1663.8447	1663.847	-0.0023	555.6222	3
			K145:NHS(1000.00)	39.58	69.15	1878.9402	1878.9377	0.0025	940.4774	2
137	145	NN(+.98)FYPREAK	free	22.25	44.5	1138.5406	1138.5407	-1E-04	570.2776	2
137	148	NNFYPREAKVQW	free	30.54	82.11	1550.7624	1550.763	-0.0006	776.3885	2
			K145:NHS(1000.00)	38.41	71.68	1765.8554	1765.8536	0.0018	883.935	2

138	148	NFYPREAKVQW	free	30.69	39.63	1436.7208	1436.7201	0.0007	719.3677	2
			N138:Deamidation:44.79;K145:NHS(1000.00)	41.71	20.92	1652.7958	1652.7947	0.0011	827.4052	2
			K145:NHS(1000.00)	38.73	61.87	1651.8116	1651.8107	0.0009	826.9131	2
140	148	YPREAKVQW	free	26.45	25.22	1175.6068	1175.6087	-0.0019	588.8107	2
143	148	EAKVQW	K145:NHS(1000.00)	38.77	22.63	974.4826	974.4821	0.0005	488.2486	2
149	173	KVDNALQSGNSQESVTEQDSKDSTY	free	23.48	81.66	2729.2308	2729.2264	0.0044	910.7509	3
			K169:NHS(68.03)	30.61	68.1	2944.3274	2944.317	0.0104	1473.171	2
			K149:NHS(1000.00);K169:NHS(1000.00)	36.85	29.29	3159.4101	3159.4076	0.0025	1054.144	3
			K149:NHS(63.21)	30.25	79.85	2944.3251	2944.317	0.0081	982.449	3
153	173	ALQSGNSQESVTEQDSKDSTY	free	22.87	89.76	2273.0014	2272.9931	0.0083	1137.508	2
155	173	QSGNSQESVTEQDSKDSTY	free	20.64	68.67	2088.8774	2088.872	0.0054	1045.446	2
156	173	SGNSQESVTEQDSKDSTY	free	20.56	73.81	1960.8152	1960.8134	0.0018	981.4149	2
			K169:NHS(1000.00)	30.65	90.07	2175.9094	2175.904	0.0054	1088.962	2
159	173	SQESVTEQDSKDSTY	free	20.41	85.09	1702.72	1702.717	0.003	852.3673	2
180	186	TLSKADY	free	19.25	27.08	796.3966	796.3967	-1E-04	399.2056	2
180	189	TLSKADYEKH	K188:NHS(81.79)	25.81	67.29	1405.6832	1405.6837	-0.0005	703.8489	2
			K183:NHS(1000.00);K188:NHS(1000.00)	36.05	63.97	1620.7738	1620.7743	-0.0005	811.3942	2
180	192	TLSKADYEKHKVY	K188:NHS(1000.00);K190:NHS(120.29)	35.35	33.07	2011.0034	2011.001	0.0024	1006.509	2
182	189	SKADYEKH	K188:NHS(120.29)	22.64	35.76	1191.5512	1191.552	-0.0008	596.7829	2
187	192	EKHKVY	K188:NHS(28.86)	24.21	29.4	1017.5234	1017.5243	-0.0009	509.769	2
202	209	SSPVTKSF	free	22.37	51.31	851.4386	851.4389	-0.0003	426.7266	2
			K207:NHS(1000.00)	37.41	57.79	1066.5288	1066.5295	-0.0007	534.2717	2
			Heavy chain_chymotrypsin							
Start	End	Peptide	PTM	RT	-10lgP	MWexp(Da)	MWthe(Da)	Error(Da)	m/z	z
1	11	EVQLVESGGGL	free	32.88	54.63	1086.557	1086.5557	0.0013	544.2858	2
			E1:NHS(1000.00)	46.76	43.91	1301.646	1301.6463	-0.0003	651.8303	2
1	18	EVQLVESGGGLVQPGGSL	free	36.56	104.27	3447.7744	3447.7744	0	1724.8945	2
			E1:NHS(1000.00)	47.77	52.48	1940.0014	1939.9851	0.0163	971.008	2
28	33	NIKDTY	free	18.16	34.46	752.3708	752.3705	0.0003	377.1927	2
37	47	VRQAPGKGLEW	free	27.13	96.04	1239.6738	1239.6724	0.0014	620.8442	2
37	47		K43:NHS(1000.00)	37.69	94.23	1454.763	1454.763	0	728.3888	2
39	47	QAPGKGLEW	free	29.69	43.9	984.5022	984.5029	-0.0007	493.2584	2
			K43:NHS(1000.00)	41.21	46.54	1199.5936	1199.5935	1E-04	600.8041	2

61	68	ADSVKGRF	free	17.79	39.76	878.457	878.461	-0.004	440.2358	2
			K65:NHS(1000.00)	32.71	67.11	1093.551	1093.5516	-0.0006	547.7828	2
58	68	TRYADSVKGRF	K65:NHS(1000.00)	31.91	28.09	1513.7616	1513.7637	-0.0021	505.5945	3
61	67	ADSVKGR	K65:NHS(1000.00)	24.34	40.13	946.4812	946.4832	-0.002	474.2479	2
63	68	SVKGRF	K65:NHS(1000.00)	31.43	30.82	907.487	907.4876	-0.0006	454.7508	2
69	77	TISADTSKN	K76:NHS(1000.00)	28.22	63.12	1150.5452	1150.5466	-0.0014	576.2799	2
69	80	TISADTSKNTAY	free	22.4	44.37	1270.5988	1270.6041	-0.0053	636.3067	2
			K76:NHS(1000.00)	32.24	81.12	1485.6958	1485.6947	0.0011	743.8552	2
69	81	TISADTSKNTAYL	free	27.43	61.84	1383.6888	1383.6882	0.0006	692.8517	2
			K76:NHS(1000.00)	37.39	68.59	1598.78	1598.7788	0.0012	800.3973	2
111	131	GQGTLVTVSSASTKGPSVFPL	free	38.36	43.04	2032.0894	2032.0841	0.0053	1017.052	2
116	127	VTVSSASTKGPS	free	18.26	88.08	1119.577	1119.5772	-0.0002	560.7958	2
116	129	VTVSSASTKGPSVF	free	29.15	94.49	1365.715	1365.714	0.001	683.8648	2
			K124:NHS(1000.00)	38.91	80	1580.8016	1580.8046	-0.003	791.4081	2
116	131	VTVSSASTKGPSVFPL	free	35.73	98.83	1575.8532	1575.8508	0.0024	788.9339	2
			K124:NHS(1000.00)	44.29	39.84	1790.9526	1790.9415	0.0111	896.4836	2
116	134	VTVSSASTKGPSVFPLAPS	free	34.6	82.7	1830.974	1830.9727	0.0013	916.4943	2
			K124:NHS(1000.00)	42.12	35.78	2046.0734	2046.0634	0.01	1024.044	2
116	136	VTVSSASTKGPSVFPLAPSSK	free	31.12	84.09	2046.0999	2046.0997	0.0002	683.0406	3
			K124:NHS(94.08)	38.19	86.52	2261.1927	2261.1903	0.0024	754.7382	3
116	145	VTVSSASTKGPSVFPLAPSSKSTSGGTAAL	free	33.43	85.93	2791.4634	2791.4604	0.003	931.4951	3
118	131	VSSASTKGPSVFPL	free	34.86	85.25	1375.7356	1375.7347	0.0009	688.8751	2
120	129	SASTKGPSVF	free	25.89	76.72	979.4972	979.4975	-0.0003	490.7559	2
			K124:NHS(1000.00)	37.89	34.77	1194.5888	1194.5881	0.0007	598.3017	2
120	131	SASTKGPSVFPL	free	34.56	77.48	1189.6338	1189.6343	-0.0005	595.8242	2
			K124:NHS(1000.00)	43.83	44	1404.7312	1404.7249	0.0063	703.3729	2
120	136	SASTKGPSVFPLAPSSK	free	29.54	65.96	1659.8862	1659.8832	0.003	554.3027	3
			K124:NHS(112.93)	37.52	72.97	1874.9758	1874.9738	0.002	938.4952	2
120	145	SASTKGPSVFPLAPSSKSTSGGTAAL	free	32.47	65.75	2405.2482	2405.2438	0.0044	802.7567	3
122	131	STKGPSVFPL	free	34.63	57.28	1031.5644	1031.5652	-0.0008	516.7895	2
122	145	STKGPSVFPLAPSSKSTSGGTAAL	free	32.5	71.61	2247.1755	2247.1747	0.0008	750.0658	3
123	131	TKGPSVFPL	free	34.77	37.12	944.5326	944.5331	-0.0005	473.2736	2
			K124:NHS(1000.00)	44.57	28.4	1159.6252	1159.6237	0.0015	580.8199	2

124	131	KGPSVFPL	free	34.64	60.85	843.4854	843.4854	0	422.75	2
			K124:NHS(1000.00)	44.69	21.63	1058.5712	1058.576	-0.0048	530.2929	2
124	134	KGPSVFPLAPS	free	33.89	27.69	1098.6068	1098.6073	-0.0005	550.3107	2
124	136	KGPSVFPLAPSSK	free	29.46	70.51	1313.7336	1313.7343	-0.0007	438.9185	3
124	145	KGPSVFPLAPSSKSTSGGTAAL	free	32.43	69.38	2059.0938	2059.095	-0.0012	687.3719	3
130	145	PLAPSSKSTSGGTAAL	free	24.89	92.37	1443.7582	1443.7569	0.0013	722.8864	2
			K137:NHS(1000.00)	35.07	77.3	1658.8478	1658.8475	0.0003	830.4312	2
134	145	SSKSTSGGTAAL	free	17.42	45.47	1065.5288	1065.5302	-0.0014	533.7717	2
128	145	VFPLAPSSKSTSGGTAAL	K137:NHS(1000.00)	41.76	32.95	1904.9946	1904.9844	0.0102	953.5046	2
132	145	APSSKSTSGGTAAL	K137:NHS(1000.00)	31.8	45.22	1448.7118	1448.7107	0.0011	725.3632	2
137	150	STSGGTAALGC(+57.02)LVK	free	20.84	20.79	1320.6782	1320.6782	0	661.3464	2
146	152	GC(+57.02)LVKDY	free	23.86	50.89	853.4004	853.4004	0	427.7075	2
146	159	GC(+57.02)LVKDYFPEPVTV	free	39.83	83.49	1622.8076	1622.8015	0.0061	812.4111	2
146	161	GC(+57.02)LVKDYFPEPVTVSW	free	44.31	102.97	1895.9142	1895.9128	0.0014	948.9644	2
148	161	LVKDYFPEPVTVSW	free	40.43	56.64	1678.8634	1678.8607	0.0027	840.439	2
149	159	VKDYFPEPVTV	free	32.19	62.38	1292.6628	1292.6653	-0.0025	647.3387	2
149	161	VKDYFPEPVTVSW	free	43.85	90.76	1565.7796	1565.7766	0.003	783.8971	2
202	212	IC(+57.02)NVNHKPSNT	free	11.86	50.89	1282.61	1282.6088	0.0012	642.3123	2
			K208:NHS(1000.00)	25.84	64.3	1497.7012	1497.6994	0.0018	749.8579	2
222	227	SC(+57.02)DKTH	K225:NHS(1000.00)	20.25	30.3	961.3916	961.3923	-0.0007	481.7031	2
222	238	SC(+57.02)DKTHTC(+57.02)PPC(+57.02)PAPELL	K225:NHS(1000.00)	36.28	105.75	2196.9714	2196.9602	0.0112	1099.493	2
245	254	LFPPKPKDTL	free	27.33	66.66	1154.67	1154.6699	1E-04	385.8973	3
			K249:NHS(11.22)	37.87	39.25	1369.76	1369.7605	-0.0005	685.8873	2
245	254	LFPPKPKDTL	free	14.99	22.6	1154.67	1154.6699	1E-04	385.8973	3
255	263	MISRTPEVT	free	22.82	50.62	1032.5268	1032.5274	-0.0006	517.2707	2
256	263	ISRTPEVT	free	19.31	20.19	901.4864	901.4869	-0.0005	451.7505	2
256	264	ISRTPEVTC(+57.02)	free	21.66	48.43	1061.5134	1061.5175	-0.0041	531.764	2
256	278	ISRTPEVTC(+57.02)VVVDVSHEDPEVKF	free	35.15	67.5	2641.304	2641.3058	-0.0018	661.3333	4
264	278	C(+57.02)VVVDVSHEDPEVKF	free	32.14	75.96	1757.8284	1757.8295	-0.0011	586.9501	3
265	278	VVVDVSHEDPEVKF	free	31.29	91.56	1597.7996	1597.7988	0.0008	799.9071	2
			K277:NHS(1000.00)	38.79	80.03	1812.8954	1812.8894	0.006	907.455	2
289	303	NAKTKPREEQYN(+.98)STY	free	17.67	27.09	1828.8632	1828.8591	0.0041	915.4389	2
			K293:NHS(39.97	25.99	33.79	2043.9574	2043.9497	0.0077	1022.986	2
			K291:NHS(1000.00);K293:NHS(1000.00)	34.09	79.68	2259.0454	2259.0403	0.0051	1130.53	2
289	299	N(+.98)AKTKPREEQY	K291:NHS(12.81)	25.63	15.67	1578.7614	1579.65	-0.8886	527.2611	3
			K291:NHS(1000.00);K293:NHS(1000.00)	35.53	34.09	1793.8562	1793.8544	0.0018	897.9354	2
289	299	NAKTKPREEQY	K291:NHS(7.32)	23.45	23.36	1577.7801	1577.7797	0.0004	526.934	3
			K291:NHS(1000.00);K293:NHS(1000.00)	33.33	76.73	1792.867	1792.8703	-0.0033	897.4408	2
290	299	AKTKPREEQY	K291:NHS(6.59)	23.09	27.85	1463.7351	1463.7368	-0.0017	488.919	3
			K291:NHS(1000.00);K293:NHS(1000.00)	33.35	28.99	1678.8294	1678.8274	0.002	840.422	2
292	303	TKPREEQYN(+.98)STY	free	19.53	66.66	1515.6858	1515.6841	0.0017	758.8502	2
			K293:NHS(1000.00)	28.59	39.25	1730.7828	1730.7747	0.0081	866.3987	2
310	322	TVLHQDWLN(+.98)GKEY	free	31.01	64.56	1602.7671	1602.7678	-0.0007	535.263	3
			K320:NHS(1000.00)	38.78	93.6	1817.8612	1817.8584	0.0028	909.9379	2
313	322	HQDWLN(+.98)GKEY	free	27	80.65	1289.5674	1289.5676	-0.0002	645.791	2
317	322	LN(+.98)GKEY	free	17.35	32.75	723.3442	723.3439	0.0003	362.6794	2
323	328	KC(+57.02)KVSN	K323:NHS(27.16)	22.7	43.03	949.4646	949.4651	-0.0005	475.7396	2
323	329	KC(+57.02)K(+215.09)VSNK	K325:NHS(8.22)	19.59	17.86	1077.5595	1077.5601	-0.0006	360.1938	3
323	337	KC(+57.02)KVSNKALPAPIEK	K323:NHS(1000.00);K329:NHS(5.26)	35.36	54.35	2112.1371	2112.1361	0.001	705.053	3
325	337	KVSNKALPAPIEK	K325:NHS(1000.00);K329:NHS(150.63)	37.69	63.71	1823.9978	1824.0105	-0.0127	913.0062	2
326	337	VSNK(+215.09)ALPAPIEK	K329:NHS(146.50)	33.53	71.57	1480.8248	1480.8249	-1E-04	741.4197	2
328	337	NKALPAPIEK	free	21.86	67.32	1079.6334	1079.6338	-0.0004	540.824	2
			K329:NHS(108.52)	33.45	51.22	1294.7246	1294.7245	1E-04	648.3696	2
329	337	KALPAPIEK	free	21.22	48.05	965.5912	965.5909	0.0003	483.8029	2
			K329:NHS(150.89)	33.33	71.95	1180.6814	1180.6815	-1E-04	591.348	2
329	338	KALPAPIEKT	free	21.99	65.12	1066.6384	1066.6386	-0.0002	534.3265	2
			K337:NHS(112.87)	33.1	65.25	1281.7294	1281.7292	0.0002	641.872	2
329	340	KALPAPIEKTIS	free	26.33	61.6	1266.7568	1266.7547	0.0021	634.3857	2
			K337:NHS(82.59)	36.73	72.91	1481.8452	1481.8453	-1E-04	741.9299	2
329	341	KALPAPIEKTISK	free	24.05	50.73	1394.8458	1394.8497	-0.0039	465.9559	3
330	337	ALPAPIEK	free	24.88	20.62	837.4956	837.496	-0.0004	419.7551	2
330	339	ALPAPIEKTI	free	31.52	59.7	1051.6266	1051.6277	-0.0011	526.8206	2
			K337:NHS(1000.00)	42.28	15.86	1266.709	1266.7183	-0.0093	634.3618	2
330	340	ALPAPIEKTIS	free	30.29	59.93	1138.6592	1138.6597	-0.0005	570.3369	2
330	342	ALPAPIEKTISKA	K337:NHS(58.92)	38.41	58.47	1552.8818	1552.8824	-0.0006	777.4482	2
338	352	TISKAK(+215.09)GQPREPQVY	K343:NHS(27.16)	33.04	23.62	1916.0061	1916.0116	-0.0055	639.676	3
341	352	KAKGQPREPQVY	free	18	22.77	1399.755	1399.7572	-0.0022	467.5923	3
342	352	AKGQPREPQVY	free	20.07	61.63	1271.6622	1271.6622	0	636.8384	2
			K343:NHS(1000.00)	29.38	69.41	1486.7512	1486.7528	-0.0016	744.3829	2
343	352	KGQPREPQVY	free	21.94	59.93	1200.6242	1200.6251	-0.0009	601.3194	2
			K343:NHS(1000.00)	29.11	65.42	1415.717	1415.7157	0.0013	708.8658	2
342	347	AKGQPR	K343:NHS(1000.00)	21.07	22.53	870.4682	870.4671	0.0011	436.2414	2
362	368	TKNQVSL	free	21.42	56.47	788.4394	788.4392	0.0002	395.227	2
353	364	TLPPSREEMTKN	K363:NHS(1000.00)	29.78	32.24	1616.7866	1616.7828	0.0038	809.4006	2
353	368	TLPPSREEMTKNQVSL	K363:NHS(1000.00)	35.38	18.85	2044.0274	2044.0259	0.0015	1023.021	2
369	375	TC(+57.02)LVKGF	free	29.49	57.92	823.4236	823.4262	-0.0026	412.7191	2
370	375	C(+57.02)LVKGF	free	28.99	75.29	722.3788	722.3785	0.0003	362.1967	2
370	384	C(+57.02)LVKGFYPSDIAVEW	free	39.97	65.25	1782.8698	1782.8651	0.0047	892.4422	2
371	384	LVKGFYPSDIAVEW	free	40.77	76.68	1622.8354	1622.8344	0.001	812.425	2
374	384	GFYPSDIAVEW	free	44.4	75.62	1282.5882	1282.587	0.0012	642.3014	2
385	394	ESNGQ(+.98)PEN(+.98)NY	free	20.06	15.44	1152.422	1152.4207	0.0013	577.2183	2
385	394	ESN(+.98)GQPENNY	free	19.87	61.58	1151.4366	1151.4367	-1E-04	576.7256	2
395	407	KTTPPVLDSDGSF	free	30.48	104.83	1362.6676	1362.6667	0.0009	682.3411	2
			K395:NHS(1000.00)	39.61	63.53	1577.7592	1577.7573	0.0019	789.8869	2
395	408	KTTPPVLDSDGSFF	free	36.61	88	1509.743	1509.7352	0.0078	755.8788	2
			K395:NHS(1000.00)	44.59	26	1724.8242	1724.8258	-0.0016	863.4194	2
415	420	VDKSRW	K417:NHS(1000.00)	24.68	32.63	1004.5022	1004.5039	-0.0017	503.2584	2

start-end	Peptide	m/z	z	pep_score	Modification of AD (#)	RT	-10lgP	MWexp(Da)	MWthe(Da)	Error(Da)
245-254	LFPPKPKDTL	578.341	2	19.57	K249,K251	30.17	31.18	1154.6669	1154.668	-0.0011
202-212	IC(+57.02)NVN(+0.98)HKPSNT	642.804	2	18.9	K208	54.64	53.48	1283.5919	1283.593	-0.0011
105-116	EIKRTVAAPSVF	659.38	2	18.77	K107	13.27	24.29	1316.7441	1316.7452	-0.0011
353-364	TLPPSREEMTKN	701.854	2	18.12	K363	13.63	22.2	1401.6915	1401.6936	-0.0021
279-303	NWYVDGVEVHNAKTKPREEQ(+0.98)YN(+0.98)STY	758.105	4	18.3	K291,K293	50.47	22.58	3028.391	3028.392	-0.001
310-322	TVLHQ(+0.98)DWLN(+0.98)GKEY	802.881	2	19.18	K320	57.17	76.32	1603.7482	1603.7472	0.001

Supplementary Method

Experiment for payload linker synthesis and conjugation to Her-IgG-PEG-AD

A. Synthesis of DBCO-linker-payload.

1. synthesis of DBCO-PEG4-DM1

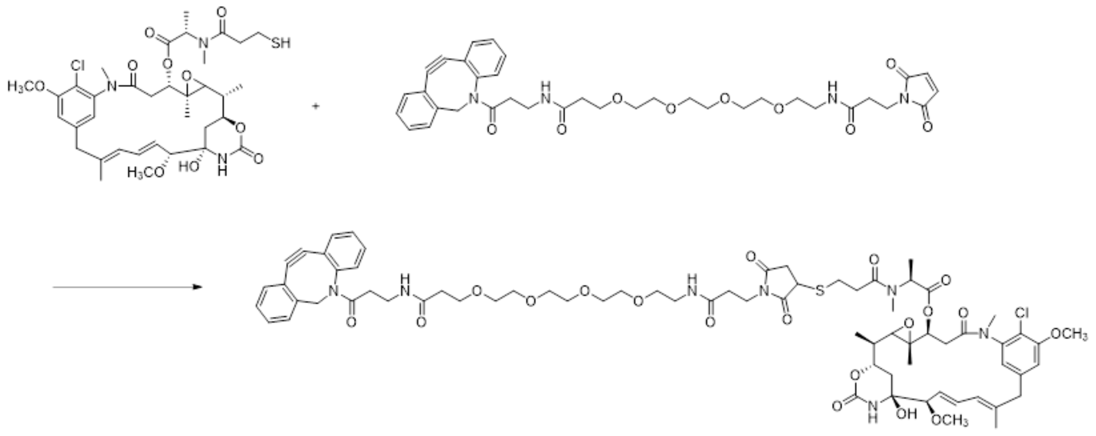

The DBCO-PEG4- maleimide and DM1 were commercial available. The DBCO-PEG4- maleimide (50mg, 1.0eq) was dissolved in acetonitrile and pH 7.4 phosphate buffer (v : v/2 : 1). DM1 (60 mg, 1.0eq) was added into the stirred solution and stirred at room temperature for 2 hours. After the reaction was complete, the reaction mixture was extracted with DCM. Then, the organic layer was washed with brine and dried over $MgSO_4$. The organic solvent was removed under reduced pressure. The residue was purified by column chromatography with methanol/dichloromethane to afford white powder (69.4 mg, 66%).

2. synthesis of DBCO-MMAF

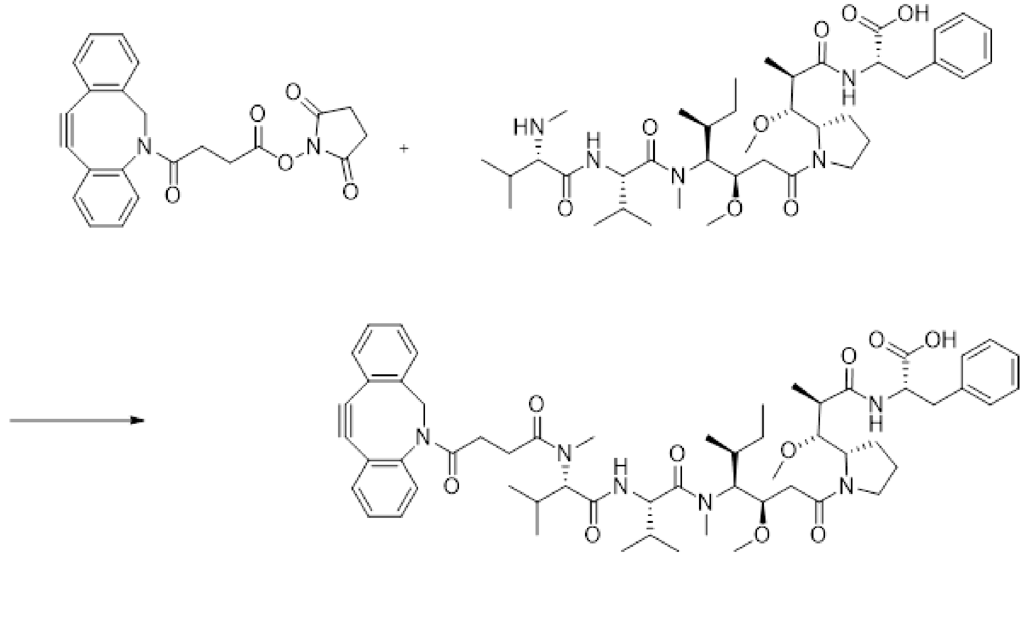

The DBCO-NHS and MMAF were commercial available. The DBCO-NHS (100mg, 1.0eq) was dissolved in 3mL DCM and 1.5mL DMF. MMAF (1.2eq) and DIPEA (2eq) was added into the stirred solution and stirred at room temperature for 16 hours. After the reaction was complete, the reaction mixture was extracted with DCM. Then, the organic layer was washed with brine and dried over $MgSO_4$. The organic solvent was removed under reduced pressure. The residue was purified by column chromatography with methanol/dichloromethane to afford white powder (63.3 mg, 25%).

B. Scheme of payload conjugation to Her-IgG-PEG-AD through click chemistry.

c). ADC conjugation

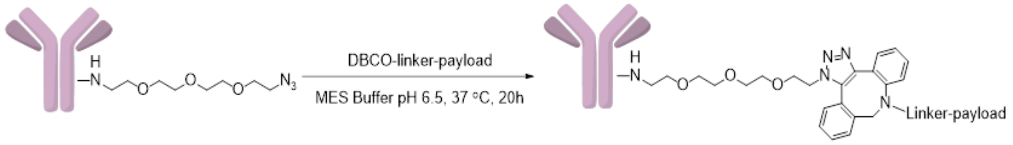

ADC 1: DBCO-linker-payload =

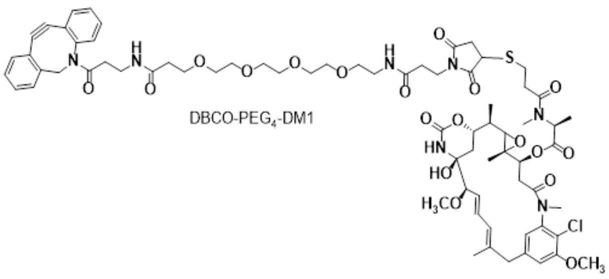

DBCO-PEG$_4$-DM1

ADC 2: DBCO-linker-payload =

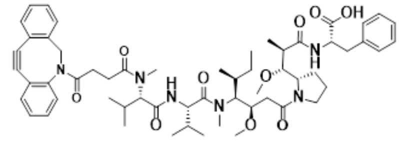

DBCO-MMAF
